# Modular transcriptional repertoire and MicroRNA target analyses characterize genomic dysregulation in the thymus of Down syndrome infants

**DOI:** 10.18632/oncotarget.7120

**Published:** 2016-02-01

**Authors:** Carlos Alberto Moreira-Filho, Silvia Yumi Bando, Fernanda Bernardi Bertonha, Filipi Nascimento Silva, Luciano da Fontoura Costa, Leandro Rodrigues Ferreira, Glaucio Furlanetto, Paulo Chacur, Maria Claudia Nogueira Zerbini, Magda Carneiro-Sampaio

**Affiliations:** ^1^ Departamento de Pediatria, Faculdade de Medicina da Universidade de São Paulo, São Paulo, SP, Brasil; ^2^ Departamento de Pediatria, Faculdade de Medicina da Universidade de São Paulo, São Paulo, SP, Brasil; ^3^ Departamento de Pediatria, Faculdade de Medicina da Universidade de São Paulo, São Paulo, SP, Brasil; ^4^ Instituto de Física de São Carlos, Universidade de São Paulo, São Carlos, SP, Brasil; ^5^ Instituto de Física de São Carlos, Universidade de São Paulo, São Carlos, SP, Brasil; ^6^ Departamento de Pediatria, Faculdade de Medicina da Universidade de São Paulo, São Paulo, SP, Brasil; ^7^ Instituto Dante Pazzanese de Cardiologia, São Paulo, SP, Brasil; ^8^ Instituto Dante Pazzanese de Cardiologia, São Paulo, SP, Brasil; ^9^ Departamento de Patologia, Faculdade de Medicina da Universidade de São Paulo, São Paulo, SP, Brasil; ^10^ Departamento de Pediatria, Faculdade de Medicina da Universidade de São Paulo, São Paulo, SP, Brasil

**Keywords:** thymus, Down syndrome, gene coexpression network, modular transcriptional repertoire, microRNA, Pathology Section

## Abstract

Trisomy 21-driven transcriptional alterations in human thymus were characterized through gene coexpression network (GCN) and miRNA-target analyses. We used whole thymic tissue - obtained at heart surgery from Down syndrome (DS) and karyotipically normal subjects (CT) - and a network-based approach for GCN analysis that allows the identification of modular transcriptional repertoires (communities) and the interactions between all the system's constituents through community detection. Changes in the degree of connections observed for hierarchically important hubs/genes in CT and DS networks corresponded to community changes. Distinct communities of highly interconnected genes were topologically identified in these networks. The role of miRNAs in modulating the expression of highly connected genes in CT and DS was revealed through miRNA-target analysis. Trisomy 21 gene dysregulation in thymus may be depicted as the breakdown and altered reorganization of transcriptional modules. Leading networks acting in normal or disease states were identified. CT networks would depict the “canonical” way of thymus functioning. Conversely, DS networks represent a “non-canonical” way, i.e., thymic tissue adaptation under trisomy 21 genomic dysregulation. This adaptation is probably driven by epigenetic mechanisms acting at chromatin level and through the miRNA control of transcriptional programs involving the networks' high-hierarchy genes.

## INTRODUCTION

Thymus provides the specialized microenvironment for the proliferation, differentiation, T-cell antigen receptor (TCR) gene rearrangement and T-cell repertoire selection [1]. The thymic microenvironment encompasses thymic epithelial cells (TEC), fibroblasts, thymic myoid cells, and bone marrow-derived accessory cells such as B lymphocytes, macrophages and dendritic cells [2]. Therefore, T-cell developmental program involves cellular processes driven by coordinate changes in the expression of hundreds of genes in the thymus [3, 4, 5, 6].

In Down syndrome (DS) [7] the gene imbalance dosage involving the Down syndrome critical region in chromosome 21 [8, 9, 10] determines a global genomic dysregulation and gene expression dysregulation domains (GEDDs) are found in discrete clusters along all chromosomes [11]. Thymic structural and functional abnormalities are among the phenotypic effects of such genomic dysregulation: DS patients present abnormal thymuses, characterized by lymphocyte depletion, cortical atrophy, and loss of corticomedullary limits. This long time recognized DS thymic abnormalities [12, 13] are not related to DS precocious senescence: DS immune system is intrinsically deficient from the very beginning [14]. This was recently confirmed by imaging studies. Sonographic thymic measurements showed that the majority of DS fetuses have smaller thymus than control [15]. Thymic-thoracic ratio (TT-ratio) evaluations obtained through ultrasound examinations showed that fetuses with trisomy 21 have a small thymus, suggesting accelerated thymic involution in utero [16].

Measuring the total number of signal joint TCR excision circles per ml blood, Bloemers et al. [17] found out that DS thymus has a decreased thymic output, concluding that “reduced thymic output, but not reduced peripheral generation nor increased loss of naive T-cells, results in the low naive T-cell numbers found in DS”. Studying the Ts65DN mouse model of DS, Lorenzo et al. [18] showed that immature thymocyte defects underlie immune dysfunction in DS and that increased oxidative stress and reduced cytokine signaling impair T-cell development. Since DS autoimmune diseases are more represented in DS, Pellegrini et al. [19] investigated phenotypic and functional alterations of natural T regulatory cells (nTreg) in DS subjects and found an over-expressed peripheral nTreg population with a defective inhibitory activity, what may be correlated with autoimmunity in DS. On the other hand, insufficient thymic expression of AIRE and peripheral antigens has been reported in DS patients [20, 21].

Global genomic dysregulation in DS also involves epigenetic mechanisms, as evidenced by the study of global changes and chromosome distribution characteristics of microRNA (miRNA) expression in lymphocytes and cord blood cells from DS children by high-throughput sequencing technology [22, 23]. It was discovered that most of the overexpressed miRNAs in DS were not Hsa21-derived. Therefore, miRNA abnormal expression in DS should be probably associated with the dysregulation of disomic genes caused by trisomy 21. Altogether, these works clearly show the importance of performing comparative global transcriptome and miRNA-target analyses in the thymic tissue of DS and karyotypic normal (CT) subjects. Such analyses are mandatory to characterize gene coexpression network (GCN) changes that could better explain the mechanisms involved in DS thymic hypofunction.

In the present work we conducted GCN and miRNA studies in thymic tissue obtained at cardiac surgery from DS and CT subjects. We constructed GCNs for DS and CT groups, separately, obtaining the networks for differentially expressed genes (DS versus CT) and also for global gene expression in each group. We adopted a network-based approach for GCN analysis - fully described in the Material and Methods section - that allows the categorization of network nodes according to distinct hierarchical levels of gene-gene connections, or node degree, and of interconnection between node neighbors, or concentric node degree [24, 25]. In a summarized way, there are three categories of high-hierarchy (HH) genes: hubs are highly connected nodes, VIPs (or Very Important Persons) have low number of connections but connect only with hubs, and high-hubs have high number of connections with highly connected nodes [24]. Moreover, we were able to identify - by using network community detection and coarse-grained community structure methods [26] - all the transcriptional modules, i.e. the distinct gene communities, present in each GCN. The use of whole tissue coupled with community structure analysis of gene interaction networks is a strategy that has been adopted for circumventing tissue microdissection [26, 27].

The integration of the above mentioned methodologies permitted: i) the visualization and analysis of GCNs for differentially expressed GO annotated genes (DE networks) and for all valid annotated transcripts (CO networks); ii) the study of interactions between all the system's constituents based on community detection, that is, on modular transcriptional repertoire analysis [reviewed in 27 and 28], leading to the identification of co-dependent gene sets involved in common functional pathways; iii) miRNA target analyses for differentially expressed miRNAs, serving to investigate epigenetic mechanisms associated to thymic GCNs and, particularly, DS genomic dysregulation.

## RESULTS

### GCN analyses

**Figure 1:**
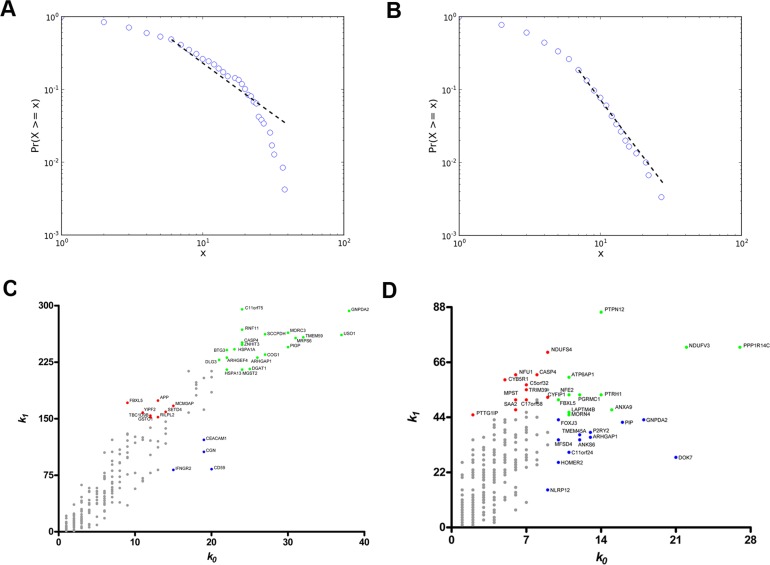
Node distribution and categorization for DE networks Kolmogorov-Smirnov test for scale free status for CT-DE (**A**) and DS-DE (**B**) networks. Scatter plots of node degree (k0) vs concentric node degree (k1) measures of GO annotated genes in CT-DE (**C**) and DS-DE networks (**D**). Hubs (blue), VIPs (red) and high-hubs (green) are identified by their gene symbols.

**Figure 2:**
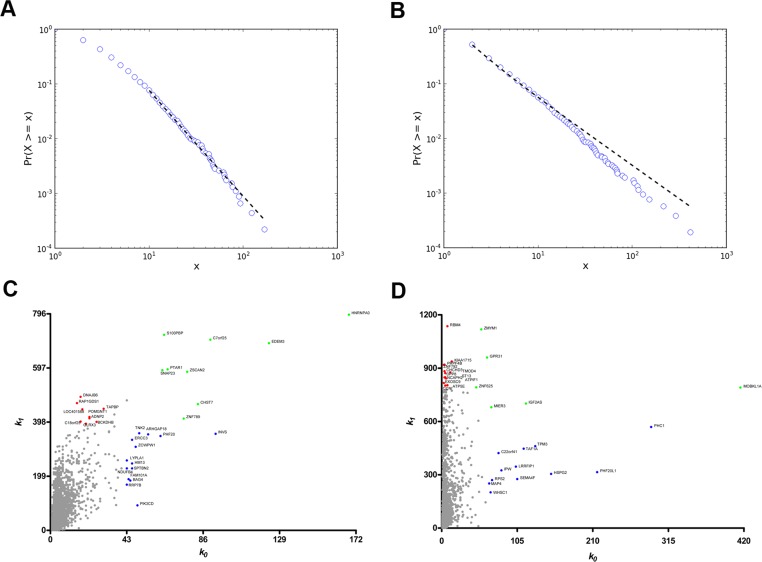
Node distribution and categorization for CO networks Kolmogorov-Smirnov test for scale free status for CT-CO (**A**) and DS-CO (**B**) networks. Scatter plots of node degree (k0) vs concentric node degree (k1) measures of GO annotated genes in CT-CO (**C**) and DS-CO networks (**D**). Hubs (blue), VIPs (red) and high-hubs (green) are identified by their gene symbols.

In the DS versus CT comparison (SAM) all differentially expressed genes (538 genes) were found to be upregulated in the DS group. Coexpression networks for differentially expressed GO annotated genes (DE networks) were constructed for CT and DS groups based on gene-gene Pearson's correlation method. A 0.968 link-strength cut-off was adopted for CT-DE network and a 0.960 link-strength cut-off was adopted for DS-DE network. The resulting DE networks had 236 genes and 908 links for CT group and 300 genes and 629 links for DS group. A 0.994 link-strength cut-off was adopted for CO networks. CT-CO presented 4,577 nodes and 8,847 edges, while DS-CO presented 5,257 nodes and 8,952 edges. All networks had scale-free node degree distribution as shown in Figure 1A and 1B for CT-DE and DS-DE networks, respectively, and in Figure 2A and 2B for CT-CO and DS-CO networks, respectively. Node categorization (hubs, VIPs, high-hubs) was accomplished using the usual node degree (*k0*) and the first neighborhood concentric node degree (*k1*), as previously described [24]. Node categorization is depicted in Figure 1C and 1D for CT-DE and DS-DE networks, respectively and in Figure 2C and 2D for CT-CO and DS-CO networks, respectively. These categorizations and the gene functions for all high-hierarchy genes (HH) in CT and DS networks (DE and CO) appear in Tables 1, 2, 3, 4 and will be further discussed in following sections. Network connectivity was found to be lower in DS-DE network: 7.69 for CT-DE and 4.19 for DS-DE; and quite similar for both CO networks: 3.86 for CT-CO and 3.40 for DS-CO.

**Table 1:** Transcriptional modules (communities), HH genes, and miRNA interactions in CT-DE network

				Function in thymus	microRNAs	
Gene	Location	Comm^a^	Category		Downregulated	Upregulated
*ARHGAP1*	11p11.2	A	HHUB	T-cell development		
*BTG3*	21q21.1	A	HHUB	T-cell development	miR-548d-5p	miR-548am-5p
*CASP4*	11q22.2-q22.3	A	HHUB	T-cell development		
*COG1*	17q25.1	A	HHUB	Golgi/ER		miR-550a-5p
*MGST2*	4q28.3	A	HHUB	Stress response		**miR-15b-5p** miR-497-5p
*MRPS6*	21q22.11	A	HHUB	Mitochondrial process		
*RILPL2*	12q24.31	A	VIP	MHC-related		
*RNF11*	1p32	A	HHUB	TEC-related		miR-129-2-3p miR-550a-5p
*SCCPDH*	1q44	A	HHUB	Thymic homeostasis	miR-548d-5p	miR-548am-5p
*TMEM59*	1p32.3	A	HHUB	Golgi/ER		
*FBXL5*	4p15.32	F	VIP	Thymic homeostasis		miR-125a-5p **miR-125b-5p** **miR-150-5p**
*GNPDA2*	4p12	F	HHUB	Thymic homeostasis	miR-548d-5p	miR-548am-5p
*MCM3AP*	21q22.3	F	VIP	Cell proliferation		
*PIGP*	21q22.2	F	HHUB	T-cell development		
*SETD4*	21q22.13	F	VIP	Epigenetic control		miR-324-3p
*TBC1D9B*	5q35.3	F	VIP	Autophagy		
*USO1*	4q21.1	F	HHUB	Golgi/ER		miR-197-3p^b^ miR-23b-3p
*DGAT1*	8q24.3	G	HHUB	T-cell development		
*HSPA1A*	6p21.3	G	HHUB	Thymic microenviron.		
*YIPF2*	19p13.2	G	VIP	unknown		
*ZNHIT3*	17q12	G	HHUB	unknown		
*C11orf75*	11q21	B	HHUB	unknown		miR-23b-3p
*CEACAM1*	19q13.2	B	HUB	T-cell development		miR-30c-5p miR-30d-5p
*IFNGR2*	21q22.11	B	HUB	T-cell development		miR-30c-5p miR-30d-5p
*CD59*	11p13	C	HUB	T-cell development		let-7f-1-3p
*CGN*	1q21	C	HUB	T-cell development		**miR-125b-5p**^b^ miR-766-3p miR-125a-5p
*HSPA13*	21q11	C	HHUB	T-cell development		**miR-181a-5p**^b^ miR-200c-3p **miR-205-5p**
*ARHGEF4*	2q22	D	HHUB	T-cell development		miR-301a-3p
*DLG3*	Xq13.1	D	HHUB	T-cell development		
*MORC3*	21q22.13	D	HHUB	Epigenetic control		let-7b-3p miR-200c-3p
*APP*	21q21.3	E	VIP	Thymic microenviron.		let-7b-3p let-7f-1-3p
*GSTO1*	10q25.1	E	VIP	Stress response		

a Comm: Community

b validated miRNA-gene interaction (miRTarBase databank); ER: endoplasmic reticulum. In bold: abundantly expressed miRNAs.

**Table 2:** Transcriptional modules (communities), HH genes, and miRNA interactions in DS-DE network

				Function in thymus	microRNAs	
Gene	Location	Comm^a^	Category		Downregulated	Upregulated
*CASP4*	11q22.2-q22.3	C	VIP	T-cell development		
*FBXL5*	4p15.32	C	HHUB	Thymic homeostasis	miR-125a-5p **miR-125b-5p** **miR-150-5p**	
*FOXJ3*	1p34.2	C	HUB	Cell cycle control	let-7f-1-3p	miR-196a-5p^b^
*GNPDA2*	4p12	C	HUB	Thymic homeostasis	miR-548am-5p	miR-548d-5p
*NDUFS4*	5q11.1	C	VIP	Mitochondrial process	miR-766-3p	
*NDUFV3*	21q22.3	C	HHUB	Mitochondrial process		
*NFU1*	2p15-p13	C	VIP	Mitochondrial process	miR-625-5p	
*PGRMC1*	Xq22-q24	C	HHUB	T-cell development	miR-486-5p	
*PTPN12*	7q11.23	C	HHUB	T-cell development	miR-149-5p miR-200c-3p	
*TRIM39*	6p21.3	C	VIP	Cell cycle control	miR-140-3p	
*ATP6AP1*	Xq28	A	HHUB	Autophagy		miR-449a
*C5orf32*	5q31.3	A	VIP	Stress response		
*CYB5R1*	1q32.1	A	VIP	Stress response		
*HOMER2*	15q24.3	A	HUB	T-cell development	let-7b-3p let-7f-1-3p miR-548am-5p	miR-548d-5p
*MORN4*	10q24.2	A	HHUB	Thymic homeostasis	**miR-205-5p**	
*MPST*	22q13.1	A	VIP	Thymic homeostasis	miR-193b-3p^b^	
*PPP1R14C*	6q24.3-q25.3	A	HHUB	Cell cycle control	miR-30c-5p miR-30d-5p	
*PTRH1*	9q34.11	A	HHUB	Thymic microenviron.		
*PTTG1IP*	21q22.3	A	VIP	TEC-related		
*DOK7*	4p16.3	D	HUB	T-cell development		
*MFSD4*	1q32.1	D	HUB	Thymic homeostasis	let-7b-3p	
*NFE2*	12q13	D	HHUB	T-cell development		
*P2RY2*	11q13.5-q14.1	D	HUB	TEC-related	miR-193b-3p	
*SAA2*	11p15.1-p14	D	VIP	unknown		
*TMEM45A*	3q12.2	D	HUB	Hassall's corp. related	**miR-181a-5p**^b^	
*ANXA9*	1q21	B	HHUB	Thymic microenviron.		
*C17orf58*	17q24.2	B	VIP	Thymic microenviron.	miR-200b-5p	
*NLRP12*	19q13.42	B	HUB	unknown		
*PIP*	7q34	B	HUB	Thymic microenviron.		
*ANKS6*	9q22.33	F	HUB	unknown	miR-125a-5p **miR-125b-5p**	
*ARHGAP1*	11p11.2	F	HUB	T-cell development		
*C11orf24*	11q13	F	HUB	Golgi/ER	miR-193b-3p^b^	
*LAPTM4B*	8q22.1	F	HHUB	Autophagy	miR-625-5p	
*CYFIP1*	15q11	H	VIP	Golgi/ER	miR497-5p	

a Comm: Community

b validated miRNA-gene interaction (miRTarBase databank); ER: endoplasmic reticulum. In bold: abundantly expressed miRNAs.

**Table 3:** Transcriptional modules (communities), HH genes, and miRNA interactions in CT-CO network

				Function in thymus	microRNAs		
Gene	Location	Comm^a^	Category		Downregulated	Upregulated
*ADNP2*	18q23	B	VIP	Cell development		**miR-15b-5p** miR-497-5p
*C18orf25*	18q21.1	B	VIP	Cell development	miR-548d-5p	miR-766-3p
*ERCC3*	2q21	B	HUB	Cell development		
*HNRNPA0*	5q31	B	HHUB	Transcription control		
*SNAP23*	15q14	B	HHUB	Cell development		
*TAPBP*	6p21.3	B	VIP	MHC-related		
*TNK2*	3q29	B	HUB	TEC-related		miR-149-5p **miR-205-5p** miR-484
*ZSCAN2*	15q25.2	B	HHUB	Transcription control		
*EDEM3*	1q25	C	HHUB	MHC-related		miR-30c-5p miR-30d-5p miR-200c-3p
*LOC401588*	Xp11.23	C	VIP	Epigenetic control		
*RAP1GDS1*	4q23-q25	C	VIP	T-cell development	miR-449a	miR-29a-3p
*S100PBP*	1p35.1	C	HHUB	Cell migration		miR-23b-3p miR-30c-5p miR-30d-5p miR-200c-3p miR-361-3p miR-455-3p miR-484
*BCKDHB*	6q14.1	F	VIP	Mitochondrial process		
*GLRX3*	10q26	F	VIP	T-cell development		
*PHF20*	20q11.22-q11.23	F	HUB	T-cell development		miR-197-3p^b^ **miR-15b-5p** miR-30c-5p miR-30d-5p miR-125a-5p **miR-125b-5p** miR-145-5p miR-301a-3p miR-497-5p
*FAM101A*	12q24.31	A	HUB	unknown		
*SPTBN2*	11q13	A	HUB	Golgi/ER		miR-15b-5p miR-361-3p miR-497-5p
*ARHGAP18*	6q22.33	D	HUB	T-cell development		
*NDUFB4*	3q13.33	D	HUB	Mitochondrial process		
*BAG4*	8p11.23	E	HUB	T-cell development		**miR-15b-5p** miR-125a-5p **miR-125b-5p** **miR-181a-5p** miR-497-5p miR-766-3p
*CHST7*	Xp11.23	E	HHUB	Thymic microenviron.		miR-23b-3p
*DNAJB6*	7q36.3	H	VIP	T-cell development		
*LYPLA1*	8q11.23	H	HUB	T-cell development		miR-23b-3p miR-29a-3p
*ZCWPW1*	7q22.1	I	HUB	Epigenetic control		
*ZNF789*	7q22.1	I	HHUB	Transcription control		
*C7orf25*	7p14.1	K	HHUB	Mitochondrial process		
*POMGNT1*	1p34.1	K	VIP	T-cell development		
*INVS*	9q31	G	HUB	unknown		
*HM13*	20q11.21	L	HUB	MHC-related		miR-149-5p miR-296-5p miR-455-3p miR-625-5p
*PIK3CD*	1p36.2	Q	HUB	Antiapoptosis		miR-30c-5p miR-30d-5p miR-125a-5p **miR-125b-5p** miR-484
*RRP7B*	22q13.2	R	HUB	unknown		
*PTAR1*	9q21.12	U	HHUB	T-cell development		miR-23b-3p miR-125a-5p **miR-125b-5p** miR-129-2-3p

a Comm: Community

b validated miRNA-gene interaction (miRTarBase databank); ER: endoplasmic reticulum. In bold: abundantly expressed miRNAs.

**Table 4:** Transcriptional modules (communities), HH genes, and miRNA interactions in DS-CO network

				Function in thymus	microRNAs	
Gene	Location	Comm^a^	Category		Downregulated	Upregulated
*ATP5E*	20q13.32	A	VIP	Mitochondrial process		
*CCNI*	4q21.1	A	VIP	T-cell development	miR-149-5p	
*CHCHD3*	7q33	A	VIP	Mitochondrial process		
*GPR31*	6q27	A	HHUB	T-cell development		
*KIAA1715*	2q31	A	VIP	Golgi/ER	miR-30c-5p miR-30d-5p miR-145-5p **miR-181a-5p** **miR-550a-5p**	
*MIER3*	5q11.2	A	HHUB	Epigenetic control	miR-331-3p^b^ miR-29a-3p miR-30c-5p miR-30d-5p miR-145-5p **miR-181a-5p** miR-200c-3p **miR-205-5p** miR-301a-3p	miR-548d-5p
*MOBKL1A*	4q13.3	A	HHUB	Cell migration	**miR-181a-5p**^b^ miR-23b-3p miR-29a-3p	
*NCAPH2*	22q13.33	A	VIP	T-cell development	miR-193b-3p^b^	
*TMOD4*	1q12	A	VIP	Thymic demise		
*ZMYM1*	1p34.3	A	HHUB	Transcription control		
*ZNF625*	19p13.2	A	HHUB	Transcription control		
*ATPIF1*	1p35.3	C	VIP	T-cell development		
*EXOSC9*	4q27	C	VIP	Transcription control		
*LRRFIP1*	2q37.3	C	HUB	Transcription control	**miR-181a-5p**	
*RBM4*	11q13	C	VIP	Epigenetic control	miR-197-3p^b^	
*TAF1A*	1q42	C	HUB	Transcription control		
*TPM3*	1q21.2	C	HUB	Cell migration	miR-145-5p^b^ miR-23b-3p **miR-150-5p** miR-204-5p miR-324-3p miR-766-3p	
*PHF20L1*	8q24.22	D	HUB	Epigenetic control	miR-23b-3p miR-129-2-3p	miR-196a-5p
*SEMA4F*	2p13.1	D	HUB	Cell migration	miR-125a-5p **miR-125b-5p** **miR-181a-5p**	miR-449a
*ZNF792*	19q13.11	D	VIP	Transcription control	miR-125a-5p miR-125b-5p	
*DPP8*	15q22	E	VIP	Thymic demise		
*PHC1*	12p13	E	HUB	Epigenetic control	miR-29a-3p miR-149-5p	
*WHSC1*	4p16.3	E	HUB	Epigenetic control	miR-193b-3p^b^ miR-23b-3p **miR-181a-5p** **miR-205-5p** miR-455-3p miR-484 miR-766-3p	
*IGF2AS*	11p15.5	B	HHUB	Epigenetic control		
*HSPG2*	1p36.1-p34	F	HUB	Thymic microenviron.	**miR-15b-5p** miR-29a-3p miR-140-3p miR-455-3p miR-484 miR-497-5p	
*RPS2*	16p13.3	G	HUB	unknown		
*PRPF4B*	6p25.2	H	VIP	T-cell development	miR-23b-3p miR-484	
*MAP4*	3p21	K	HUB	T-cell development	**miR-15b-5p** miR-23b-3p miR-1267 miR-301a-3p miR-497-5p miR-766-3p	
*IPW*	15q11.2	L	HUB	Epigenetic control		
*C22orf41*	22q13.33	S	HUB	unknown		
*ST13*	22q13.2	Y	VIP	Thymic homeostasis		

a Comm: Community

b validated miRNA-gene interaction (miRTarBase databank); ER: endoplasmic reticulum. In bold: abundantly expressed miRNAs.

### Community detection

**Figure 3:**
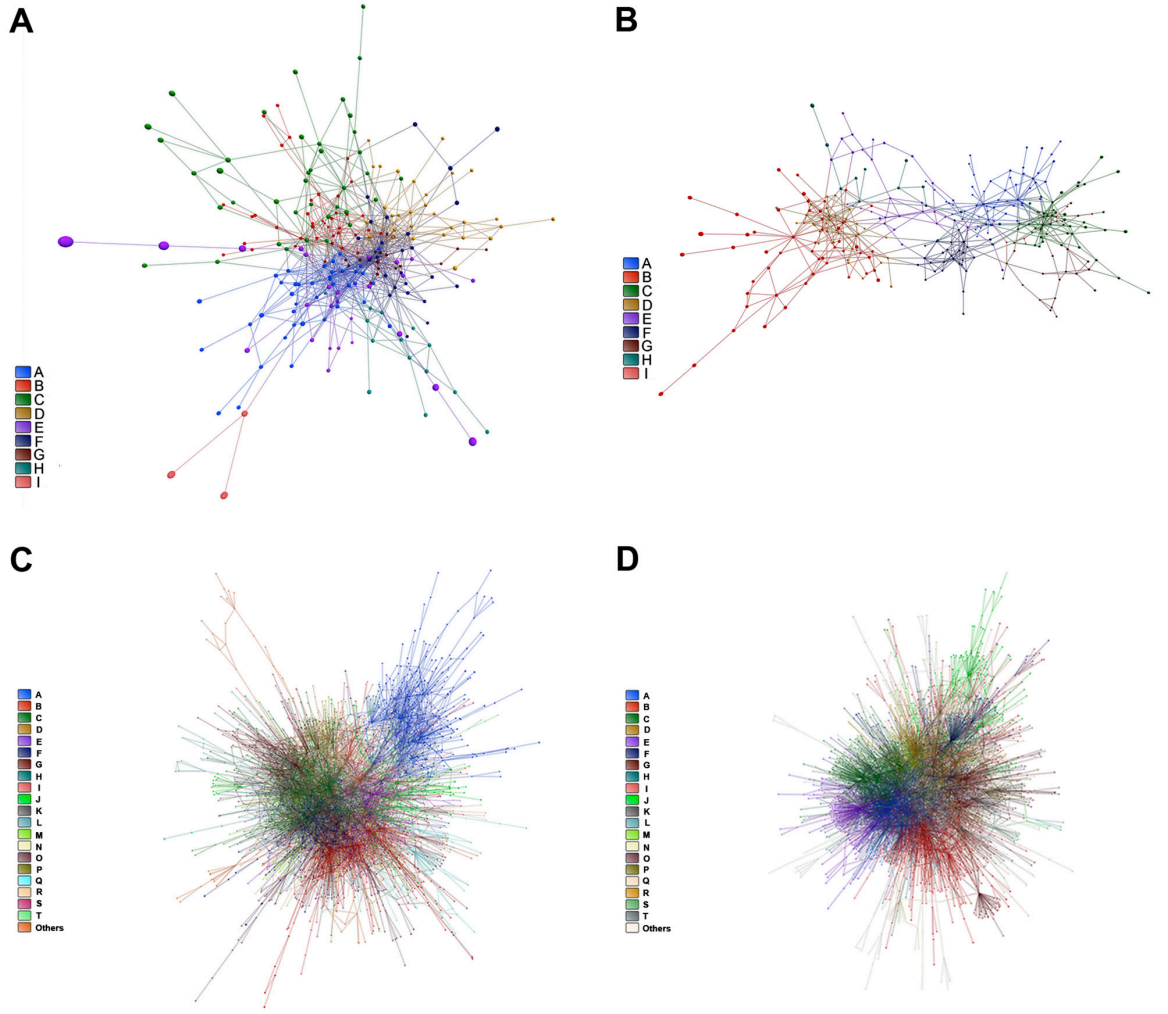
DE and CO networks and respective gene communities (modules) Network topology and community structure for CT-DE, DS-DE, CT-CO and DS-CO networks are depicted in **A**, **B**, **C** and **D**, respectively. Gene communities are distinguished by different colors and identified by the right side bar code.

An overall picture of DE and CO gene communities (modules) is depicted in Figure 3A and 3B for CT-DE and DS-DE networks, respectively and in Figure 3C and 3D for CT-CO and DS-CO networks, respectively. Different node colors identify the distinct gene communities in each network. Networks presented good quality of community structure and gene distribution. Modularity value was higher for DS-DE network: 0.705 against 0.450 for CT-DE, and quite similar between CO networks: 0.732 for CT-CO and 0.693 for DS-CO. The DE networks harbor the same number of communities (nine) but the DS network has lower connectivity and its communities are more sparsely connected, what may indicate a higher grade of dysregulation in cell's functional organization [29, 24]. The CT-CO and DS-CO networks harbor 27 and 32 communities, respectively. The number of genes per community can be found in Tables S1 and S2, for DE and CO networks, respectively. A set of simulations run with slightly different link-strength thresholds (from 0.900 up to 0.990 for DE networks, and from 0.980 up to 0.999 for CO networks) did not reveal alterations in community structures, thus indicating their robustness.

### Coarse-grained community structure analysis (DE and CO networks)

**Figure 4:**
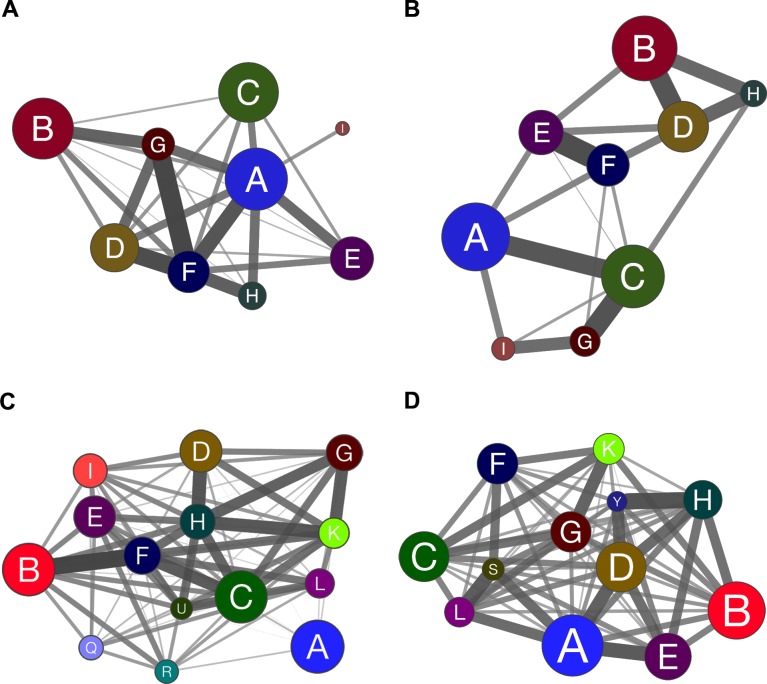
Coarse-grained community structure (CGCS) diagrams showing the relationships among gene communities obtained for DE and CO networks CGCSs are depicted for all communities of CT-DE (**A**) and DS-DE (**B**) networks. CGCSs CT-CO (**C**) and DS-CO (**D**) networks are restricted to communities harboring at least one high-hierarchy gene. The communities, identified by different colors in each CGCS diagram, are collapsed in a single node and edges connect all the communities weighted by the fraction of edges existing against all possible edges between two communities. The edge width and intensity is proportional to the connection weight of edges linking distinct communities. The node size is proportional to the number of nodes in each community.

**Figure 5:**
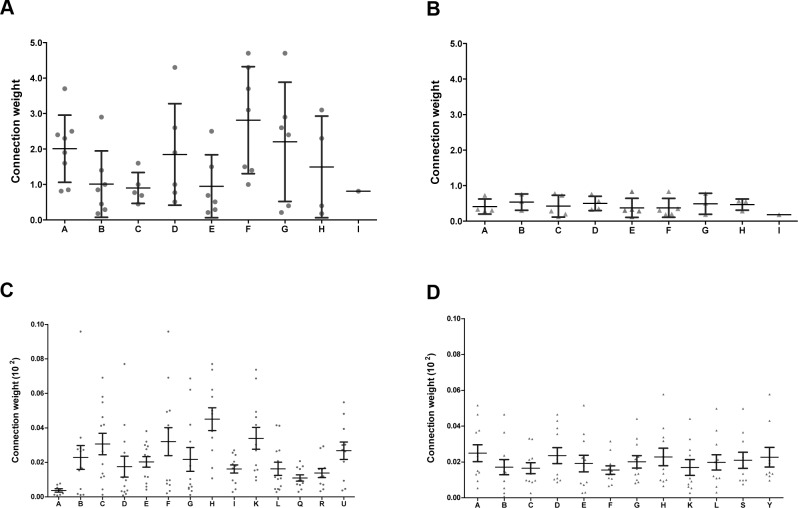
Connection weight values Connection weight values were obtained for all the constituent communities of CT-DE (**A**) and DS-DE (**B**) networks, and for the CO communities harboring at least one high-hierarchy gene, here depicted in (**C**) for CT-CO and in (**D**) for DS-CO. Dots represent the connection weights of the links centered in each community.

Coarse-grained community structure (CGCS) was obtained for each network, yielding the relationships between each community in the network (Figure 4A and 4B for DE; 4C and 4D for CO). For CO networks only communities harboring HH genes were considered in this analysis. Connection weight values for DE and CO network's communities are depicted in Figure 5 and show the overall lower connection weight of DS networks' communities. Most of the DS-CO communities also presents lower connectivity values (Figure S1) when compared to CT-CO communities. Altogether, these measures indicate a certain degree of disorganization of inter and intra modular interactions, probably due to gene dosage imbalance.

Communities having the highest node strength (total probability for their nodes to connect to distinct communities) hold the most significant functional interactions in the network [27, 30, 31]. Therefore, the subsequent analysis of gene communities in DE and CO networks was performed considering not only the gene/node hierarchy but, and principally, the networks' CGCSs.

### MicroRNA target analyses

**Figure 6:**
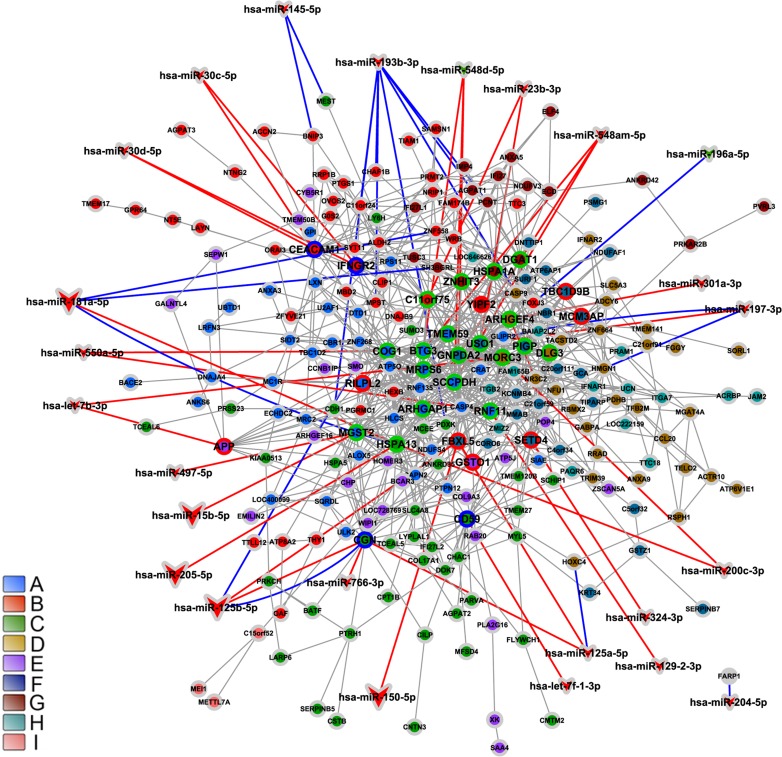
Integrative network analysis for CT-DE network CT-DE network modular transcriptional repertoire structure (communities) is depicted together with all miRNA-gene validated interactions (blue lines) and the predicted miRNA-gene interactions involving at least one HH gene (red lines). Communities are distinguished by nodes with different filled colors. Circle or vee nodes represent respectively genes or miRNA. The circles with green, blue or red border indicate respectively high-hubs, hubs or VIPs. The vees filled with red or green colors indicate respectively miRNA hyper- or hipo-expressed. Larger vees indicate abundantly expressed miRNAs. Gene-gene links are indicated by gray lines. Gene node size is proportional to node degree (*k_0_*).

We obtained 53 differentially expressed miRNAs: 47 down-regulated and 6 up-regulated in DS group. Six of these miRNAs were found to be abundantly expressed (a 30 times average increase) in CT and DS thymic tissue (Table S3). The interaction between these 53 differentially expressed miRNAs and the 538 DE genes (obtained through SAM) resulted in a miRNA-DE genes interaction graph containing 219 DE genes and 53 miRNAs, summing up 455 interactions. A total of 10 miRNAs have experimentally validated gene-miRNA interactions, whereas the predicted interactions encompassed all differentially expressed miRNAs. Tables 1 and 2 contain brief gene function descriptions for all HH genes in DE networks and: i) all miRNA-gene validated interactions; ii) predicted miRNA-gene interactions involving at least one HH gene. The gene coexpression networks for CT-DE and DS-DE groups are displayed in Figure 6 (CT-DE network) and 7 (DS-DE network). Code colors identify the distinct gene communities. Hierarchy-categorized selected nodes are identified by their corresponding GO gene symbols; node border colors indicate hubs (blue), VIPs (red) or high-hubs (green). MiRNA-gene interactions are shown for high- and low-hierarchy genes: larger vees correspond to abundantly expressed miRNAs; the blue lines indicate validated interactions whereas predicted interactions are indicated by red lines.

MiRNA interactions with CO network genes (CT-CO and DS-CO) were investigated only for high-hierarchy genes (HH) employing miRTarBase (experimentally validated miRNA-target gene interaction database) and mirPath (predicted miRNA-target gene interaction database). Accordingly, HH subnetworks were constructed for CT and DS groups. The CT-CO HH subnetwork (Figure 8A) had 39 nodes and 54 edges, whereas the DS-CO network encompassed 44 nodes and 59 edges (Figure 8B). Code colors and symbols were the same described above for DE networks. In the CT-CO subnetwork, a total of 25 miRNAs were found to interact with 14 HH genes. In the DS-CO subnetwork we identified 29 miRNAs interacting with 15 HH genes (Figure 8B). Again, larger vees indicate abundantly expressed miRNAs, the blue lines indicate validated interactions, and predicted interactions are indicated by red lines. Tables 3 and 4 contain the description of gene functions and miRNA interactions (validated and predicted) for all HH genes in CO subnetworks.

### Gene communities and microRNA-gene interactions in CT and DS networks

Here we summarize the biological functions of the HH genes - hubs, VIPs and high- hubs - found in each of the DE and CO networks' communities. Since these genes play an essential role in keeping network's structure and functions [29, 32, 33], their interactions with differentially expressed miRNAs are also considered in this section. Tables 1, 2, 3, 4 show community distribution, biological functions/products, and miRNA interactions assigned for the HH genes in each network.

### CT-DE network

In CT-DE, communities A and F (Figure 6 and Table 1) encompass most of the HH genes. Interestingly, A and F harbor four genes that also appear as HH genes in DS-DE network. Two of these genes, *FBXL5* [34, 35] and *GNPDA2* [36], both in community F, have biological functions associated to the maintenance of thymic microenvironment (iron homeostasis, ROS sensing, lipid metabolism). The two other HH genes shared with DS-DE network belong to community A: one is *ARHGAP1*, a RhoGTPase activating protein [37, 38], and the other is *CASP4*, which is involved in thymic lymphopoiesis [39, 40].

Community A encompasses ten HH genes and nine of these genes are high-hubs (Table 1), thus indicating their relevance for network functioning and stability [33, 41]. Interestingly, two of these high-hubs are located in DSCR, the Down Syndrome Critical Region (21q21- 21q22.3) of chromosome 21 (HSA21) [10]. One is *BTG3*, which codes for a member of the anti-proliferative BTG/Tob protein family known as ANA or BTG3 [42]. This gene and *BTG2*, another BTG/Tob family member, act together in the regulation of stage-specific proliferation of developing thymocytes [43]. The other high-hub is *MRPS6*, a gene that encodes the mitochondrial ribosomal protein S6, a component of the oxidative phosphorylation system (OXPHOS), the main source of T-cell's energy under resting conditions [44]. OXPHOS is one of the canonical cellular pathways affected by changes in gene expression levels with thymocytes age [45]. These two genes are known to have altered expression in Down syndrome [46, 47]. All the other genes in community A are also associated to thymic cell development/selection, or to thymic cell survival and apoptosis pathways, as described below.

*ARHGAP1* (aliase *RhoGAP*), codes for a Rho GTPase activating protein. Rho GTPase and RhoGAP are essential for cell motility [48]. Rho GTPase has critical regulatory roles in thymus development, such as thymocyte proliferation and survival, and thymic egress [37, 38]. Additionally, *RhoGAP* deletion is associated with thymic cancer [49]. *CASP4* codes for caspase-4, an apoptosis-related cysteine peptidase which is also an activator of caspase-1[39]. The activation of caspase-1 decreases thymic lymphopoiesis [46]. *RILPL2*, the only VIP in community A, is involved regulating lysosomal related organelles (LRO) morphology and MHC-II presentation [50]. *COG1* acts on Golgi-associated processing of glycoconjugates and intra-Golgi trafficking [51, 52]. *SCCPDH* codes for saccharopine dehydrogenase, an enzyme involved in lysine metabolism [53] and, by extension, in thymic homeostasis [54]. *RNF11*gene product is the RING finger protein 11, which binds to Smad4 and enhances Smad4-dependant TGF-β signaling [55]. TGF-β signaling has a pivotal role in the regulation of medullary thymic epithelial cell development [56]. *MGST2* (aliase *GST2*) codes for glutathione S-transferase 2, a molecule involved, via S-glutathionylation, in response to oxidative stress and in cell survival pathways [57, 58, 59]. And, lastly, *TMEM59*, a gene coding for a ubiquitously expressed Golgi-associated protein involved in selective autophagy [60], and also in the glycosylation, cell surface expression, and secretion of APP, the amyloid precursor protein [61]. APP is secreted by thymic stromal cells [62].

The high-hierarchy genes in community F comprise four VIPs and three high-hubs (Table 1), whose functions are briefly described here. One of the VIPs, *FBXL5*, and the high-hub *GNPDA2* are related to thymic microenvironment and were already commented above. Two HH genes in this community are related to cell proliferation and apoptosis. One is *MCM3AP*, a VIP that codes for the MCM3 (minichromosome maintenance protein 3) binding protein. MCM3AP interacts with the germinal center-associated protein (GANP) and the glucocorticoid receptor in order to regulate cell proliferation [63]. The other is the high-hub *USO1*, which is involved in ER-to Golgi transport, regulating mitosis progression and apoptosis [64]. The high-hub *PIGP* encodes the GPI (glycosylphosphatidylinositol)-anchored semaphorin7A, a protein that regulates T-cell development, especially positive selection [65, 66]. *SETD4* is a VIP that codes for a cytosolic and nuclear lysine methyltransferase related to histone lysine methylation [67]. Histone methylation serves to regulate chromatin and gene expression. *TBC1D9B* is a high-hub and codes for a Rab GTPase-activating protein involved in the regulation of endocytic and autophagy pathways [68]. Autophagy is an essential process for negative and positive thymocyte selection, for promoting Treg and iNKT cell differentiation, and for thymocyte survival as well [69, 70]. It is also noteworthy that three of these genes - *MCM3AP*, *PIGP* and *SETD4* - are located in DSCR (Table 1). Moreover, *PIGP* was found to be overexpressed in the fetal cortex brain of Down syndrome subjects [71].

Overall, most of the HH genes in communities A and F have relevant roles in thymus functioning and microenvironment. The very fact that five of these genes are found to be located in DSCR/HSA21 indicates that chromosome 21 dysregulation may impact thymus development and functioning. This is confirmed by the finding that three additional DSCR genes and one HSA21 (21q11) gene appear among the HH genes in the other CT-DE communities (Table 1), as described below. Community F has the highest connection weight in CT-DE network and community A has the third one (Figure 5A).

Community C harbor only three HH genes: two of them, *CD59* and *CGN*, are hubs. *CD59* codes for a complement regulatory protein located on Hassall's corpuscles and medullary epithelial cells [72]. CD59 is putatively involved in the thymic selection of T regulatory cells [72, 73]. *CGN* codes for cingulin, a tight junction protein present in Hassal's corpuscles [74]. Cingulin regulates RhoA signaling [75], which has an essential role in thymocyte development [76]. The third HH gene in community C is the high-hub *HSPA13* (located on 21q11) which codes for the heat shock protein family member 13, known as SCTH. One of the SCTH functions is to sensitize cells to tumor necrosis factor-related apoptosis-inducing ligand (TRAIL)-induced apoptosis [77]. TRAIL is a mechanism underlying thymic negative selection [78]. Therefore, all these three genes act on thymus medullar area.

Community B has three HH genes. Two of these genes - *CEACAM1* and *IFNGR2* - are hubs and exert known functions in thymus. *CEACAM1* encodes a type I-transmembrane glycoprotein which is a coinhibitory receptor for TCR-CD3 complex signaling [79]. *IFNGR2* codes for the IFN-γ receptor 2, a molecule involved in the migration of mature thymocytes [80]. This gene is located in the DSCR (21q22.11). The third gene is *C11orf75* (aliase *SMCO4*), a high-hub coding for the single-pass membrane protein (HGNC 24810) with coiled domains 4, whose functions in thymus are yet unknown.

Community D has three high-hubs. *MORC3* codes for a highly conserved nuclear protein that is an epigenetic regulator associated with senescence and p53 regulation [81, 82]. This gene is located in the DSCR (21q22.13). *ARHGEF4* codes for Asef, a guanine-nucleotide exchange factor (GEF) which is specific for Cdc42 [83], a Rho GTPase critically involved in thymopoiesis [84]. Finally, there is *DLG3*, a gene coding for a membrane-associated guanylate kinase (MAGUK) [85] which recruits Nedd-4 ubiquitin ligases involved in key aspects of TCR signaling and T-cell functioning [86]. This community has the fourth highest connection weight value among the eight CT-DE communities harboring HH genes.

Community E has only two HH genes and both are VIPs. *APP* (located in DSCR, 21q21.3) codes for the amyloid precursor protein, a hallmark of Down syndrome [87], whose overexpression severely affects thymic functions. *GSTO1* codes for a glutathione S-transferase involved in oxidative stress response and detoxification processes [58, 88].

Community G is the only CT-DE community with no HH genes in HSA21. This community has four HH genes, whose functions are summarized here. *DGAT1* is a high-hub involved in diacylglycerol metabolism (DAG). DAG attenuates T-cell receptor signaling and ensures T-cell passage along check-points during thymocyte differentiation [89]. *HSPA1A*, also a high-hub, codes for a HSP70 chaperone that accelerates protein translocation and the unfolding of stable protein aggregates [90, 91]. *YIPF2*, a VIP, codes for a member of the Yip1 family of proteins. Proteins of this family are mostly involved in ER-to Golgi membrane transport, but the specific function of human YIPF2 is still unkown [92]. The remaining HH gene in this community is the high-hub *ZNHIT3*, which codes for a C/D small nucleolar ribonucleoprotein assembly factor [93], hitherto without a defined function in thymus. Community G has the second highest connection weight value in CT-DE network (Figure 5A).

An overview of CT-DE communities (Table 1) shows that many HH genes are related to relevant thymic functions, thus reflecting the correlation between transcriptional modules, or network communities, with thymic functional areas. For instance: community C is related to Hassall's corpuscle and medullar area; communities A and F are principally related to thymocyte development and thymic microenvironment; communities B and D are linked to thymocyte development. Moreover, the communities with highest connection values harbor most of the HH genes related to relevant thymic processes, chiefly linked to thymic pathways and thymus microenvironment. The correspondence between transcriptional and functional modules in biological systems has been found in other genomic studies of immunological processes using whole tissues [revised in 27].

### MicroRNA target interactions in CT-DE

In CT-DE network the communities A, F, and C concentrate most of the interactions between HH genes and differentially expressed miRNA interactions. Most importantly, four genes in these communities are the only ones in the network to have interactions with differentially expressed miRNAs that are abundantly expressed in thymic tissue (Table 1, Figure 6). It is widely accepted that miRNAs give robustness to biological processes by buffering fluctuations in gene expression and/or attenuating aberrant transcripts [94]. Most of the genes in those three communities fulfill important tasks for thymic functioning, such as the ones in community C, which are related to Hassall's corspuscle and thymic medullar area. Three out of the six abundantly expressed miRNAs interacting with CT-DE network genes are found in this community - two of them exclusively. Additionally, the two genes interacting with abundantly expressed miRNAs in communities A and F, namely *MGST2* and *FBXL5* have important roles in maintaining thymic homeostasis. *FBXL5* is also a high-hub in DS-CT network, where it interacts with the same abundantly expressed miRNAs, miR-125-5p (a HSA21-derived miRNA) and miR-150-5p. Interestingly, community A harbors all the interactions involving the miR-548 family, which is one of largest miRNA families in human genome and some of its members may form miRNA-miRNA duplexes [95]. Finally, it is also noteworthy that in CT-DE communities most of the miRNA-gene interactions occur with high-hubs, the gene category that contributes most to the network robustness [33, 24].

### DS-DE network

**Figure 7:**
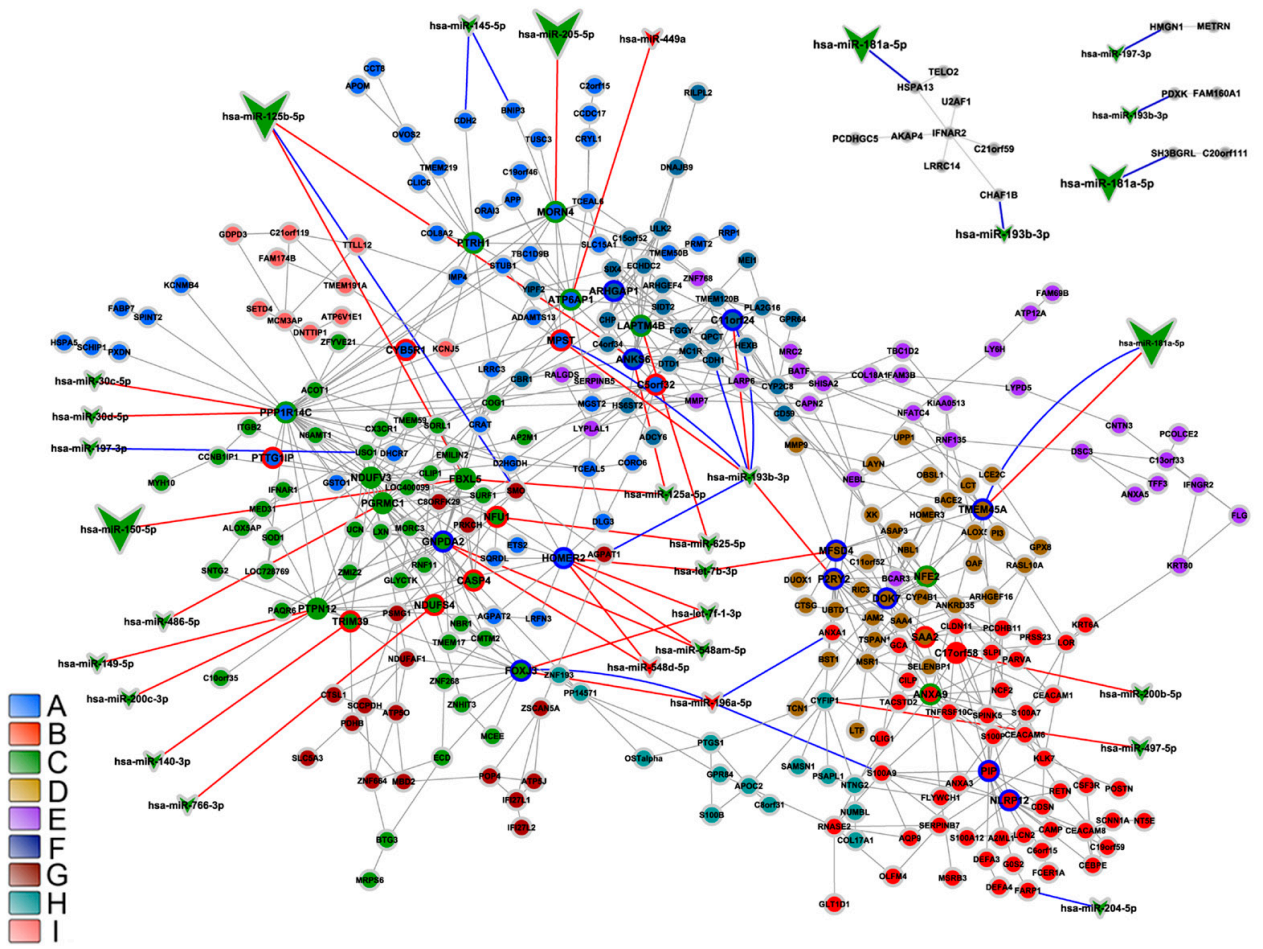
Integrative network analysis for DS-DE network DS-DE network modular transcriptional repertoire structure (communities) is depicted together with all miRNA-gene validated interactions (blue lines) and the predicted miRNA-gene interactions involving at least one HH gene (red lines). Communities are distinguished by nodes with different filled colors. Circle or vee nodes represent respectively genes or miRNA. The circles with green, blue or red border indicate respectively high-hubs, hubs or VIPs. The vees filled with red or green colors indicate respectively miRNA hyper- or hipo-expressed. Larger vees indicate abundantly expressed miRNAs. Gene-gene links are indicated by gray lines. Gene node size is proportional to node degree (*k_0_*).

The DS-DE communities (Figure 7 and Table 2) depict thymus functioning under Down syndrome genomic dysregulation. Yet, only two out of 34 HH genes in DS-DE network locate on DSCR in HSA21: *PTTG11P* [96] and *NDUFV3*, in communities A and C, respectively. *NDUFV3*, which codes for a NHDH-ubiquinone oxidoreductase complex subunit, is overexpressed in Down syndrome and contributes to DS phenotype [97, 98, 99]. However, three other genes in this network - *CYB5R1* [100] and *ATP6AP1* [101] in community A and *NFE2* [102] in community D - are well known DS biomarkers with increased expression in DS subjects [103, 104, 105], although not located on HSA21. In DS-DE network most of the HH genes are concentrated in communities A and C (Table 2).

In community A, three out of its nine genes are related to processes involving thymic epithelial cells and thymocyte development, as follows. *ATP6AP1*, a high-hub, is involved in macroautophagy [106]. Macroautophagy substrates are loaded onto MHC class II of medullary thymic epithelial cells for central tolerance [101]. *HOMER2*, a hub, codes for member of the Homer family of scaffolding proteins which is involved, via NFAT (nuclear factor of activated T-cell) and calcineurin interactions, in thymocyte development [107, 108]. *PTTG1IP*, a VIP, codes for the pituitary tumor-transforming 1 (PTTG1) interacting protein and its coexpression with PTTG1 leads to the transcriptional activation of basic fibroblast growth factor, an inducer of thymic epithelial cell differentiation [96].

The other six genes in community A are mostly related to stress tolerance and cell survival. Three of these genes are VIPs: *C5orf32* that encodes a cysteine-rich transmembrane module with a role in stress tolerance across eukaryotes [109]; *CYB5R1*, which codes for the cytochrome b5 reductase, is overexpressed in DS and its increased expression is associated with higher oxidative stress [100]; and *MPST*, which codes for mercaptopyruvate sulfurtransferase, an antioxidant protein [110]. The three high-hubs are: *PTRH1*, coding for a peptidyl RNA hidrolase involved in the rapid clearing of peptidyl-t-RNA, thus preventing cell death [111]; *PPP1R14C*, that codes for a protein phosphatase involved cell cycle and metabolism control [112]; and *MORN4* that codes for a MORN-repeat containing protein regulating Ca2+ homeostasis [113, 114].

In community C, five out of the ten HH genes are related to thymic homeostasis, as follows. The high-hub *FBXL5* and the VIP *GNPDA2* - also HH genes in CT-DE network (Table 1) - are associated, respectively, to iron homeostasis [34] and ROS sensing [40], and to lipid and glucose metabolism [36]. The VIPs *NDUFS4* [115] and *NFU1* [116], as well as the high-hub *NDUFV3*, a DS biomarker [99], are members of mitochondrial respiratory chain complex I. The other five genes are mostly linked to thymocyte development and cell proliferation control. *CASP4*, a VIP, is a negative regulator of thymopoiesis [39, 36] also present in CT-DE (Table 1). *FOXJ3*, a hub, codes for a forkhead transcriprion factor and acts on cell cycle control [117]. *PTPN12* is a high-hub and codes for a tyrosine phosphase that acts as a regulator of T-cell antigen receptor function during thymocyte development [118]. *TRIM39*, a VIP, codes for the RING finger protein 39 (RNF23) and is an antiapoptotic gene [119]. Finally, there is *PGRMC1*, a high-hub, that encodes the progesterone receptor membrane component 1, an adaptor protein for steroid receptors [120], which may be involved in thymic output regulation [121].

Each of the other communities in DS-DE network can be associated to a particular set of functions or processes in the DS thymus. Accordingly, in community D, most of the HH genes are related to thymocyte differentiation and/or to thymic medullar area. This community includes one high-hub, one VIP and four hubs. *NFE2* is the high-hub and codes for the transcription factor nuclear factor erythroid 2 (NF-E2). This gene is overexpressed in DS [105] and NF-E2 may act, via DNase I hypersensitive site HS5 [122], in the induction phase of IL-4 expression in thymic CD4 T-cells [102]. *SAA2* is the VIP and codes for the acute phase protein serum amyloid A2 (SAA). SAA proteins induce mitogenic signals in peripheral regulatory T-cells via monocyte activation [123], but their function in thymus is still unknown. Two of the four hubs - *TMEM45A* and *MFSD4* - have functions clearly related to thymic medullar area. *TMEM45A* codes for a transmembrane protein (TMEM) linked to keratinization and found in Hassall's corpuscles [124]. *MFSD4* encodes the transmembrane transporter of MFS (small solute transport) proteins. Gene network analysis revealed that *MFSD4* takes part, via *PDE3B* (phosphodiesterase 3B gene), in Foxp3 regulation and Treg homeostasis [125]. *DOK7* codes for the docking protein-7, a recruiting protein for Nck1/2 adapter proteins [126]. Nck1/2 proteins enhance TCR signaling strength, thus fine-tuning the threshold of thymocyte selection [127, 128]. *P2RY2* gene product is the P2Y2 purinergic receptor, widely expressed by thymic epithelial cells [129, 130, 131] and involved in the phagocytic clearance of apoptotic thymocytes [132].

Community B has four HH genes. Two, *ANXA9* and *PIP*, are expressed in epithelial cells and associated to thymic microenvironment. *ANXA9* is a high-hub and codes for annexin 9, a periplakin and keratin 8 interacting partner [133]. Keratin 8 is a key promoter of thymic epithelium integrity [134]. *C17orf58* is a VIP and codes for a GDP-D-glucose phosphorylase involved in glycoprotein biosynthesis and metabolic repair [135]. *PIP* is a hub that codes for the prolactin-inducible protein, a regulator of integrin signaling and fibronectin cleavage [136], thus playing a role in keeping thymic microenvironment [137]. Lastly, *NLRP12* is a hub that codes for the nucleotide-binding domain and leucine-rich repeat containing receptor (NLRP) 12, which is negative regulator of NF kappa-β signaling [138]. NLRP12 has a quite clear role in the negative control of peripheral T-cell response, but its function in thymic pathways remains to be clarified [139].

Community F also has four HH genes: two of these genes are related to thymocyte selection and one to anterograde trafficking in Golgi apparatus. *LAPTM4B*, a high-hub, encodes a lysosomal-associated transmembrane protein involved in autophagy initiation [140, 141]. Autophagy, as mentioned before, is an essential process for thymocyte selection and T-cell differentiation [69, 70]. The hub *ARHGAP1* (a high-hub in DS-DE, Table 1) codes for a RhoGTPase exerting relevant roles in thymocyte development, as commented above [37, 38]. *C11orf24* is also a hub and codes for a type I membrane protein that cycles between the Golgi apparatus and the plasma membrane [142]. The third hub in this community, *ANKS6*, codes for a protein containing nine ankyrin repeats and a SAM domain [143]. ANKS6 is a central protein in the nephronophthisis module [143], but its function in the thymus remains unknown.

Community H harbors only one HH gene: *CYFIP1* is a VIP that codes for a clathrin heavy chain binding protein associated to the trans-Golgi network (TGN) [144].

A general picture of DS-DE reveals that almost half of the HH genes in this network (13 out of 34) are related to roles in thymic homeostasis and cell survival. Thymocyte development and Treg and thymic medullar processes are also well represented gene functions. This scenario is compatible with compensatory processes elicited by trisomy 21 genomic dysregulation. The profile of miRNA target interactions in DS-DE network, described below, corroborates this interpretation. All DS-DE network communities present very low connection weights (Figure 5B) when compared to the values found for CT-DE communities. These figures indicate that trisomy 21 dysregulation led to an overall diminished connectivity in the DS-DE network. Indeed, comparatively to CT-DE network, the DS-CT network present low connectivity and high modularity, what is evident comparing Figure 2A and 2B. The CGCS diagrams (Figure 4) also depict a relative “paucity of connections” between DS-DE communities vis-à-vis CT-DE (Figure 4A and 4B). As a whole, this scenario indicates a comparatively reduced interaction between DS-DE transcriptional modules what could underlie the early-onset thymic dysfunction in DS subjects. The pie chart depicted in Figure 9 shows a compative view of HH gene functional profiles in CT-DE (Figure 9A) and DS-DE (Figure 9B) network.

### MicroRNA target interactions in DS-DE

In DS-DE four out of the six network communities harbor HH genes interacting with differentially expressed miRNAs that are abundantly expressed in thymic tissue (Table 2, Figure 7). However, these interactions target just one gene per community. Two of these genes - *FBXL5*, also a high-hub in CT-DE, and *MORN4* - are involved in thymic homeostasis and are located in communities C and A, respectively. The other two interactions with abundantly expressed miRNAs involve *TMEM45A*, a gene related to Hassall's corpuscles and located in community D, and *ANKS6*, located in community F, whose function in thymus is not yet determined. Most of the miRNA-gene interactions occur in communities A and C that contain the majority of the HH genes in DS-DE network. These communities also harbor the greater part of the genes involved in thymic homeostasis and cell survival, two distinctive functional features of DS-DE. The vast majority of the miRNAs interacting with HH genes in DS-DE network are down-regulated, what is well-matched with the overall upregulation of differentially expressed genes in DS group. This scenario probably depicts the outcome of genomic dysregulation caused by the trisomy 21. This subject will be further expanded in the Discussion section.

### CO networks

**Figure 8:**
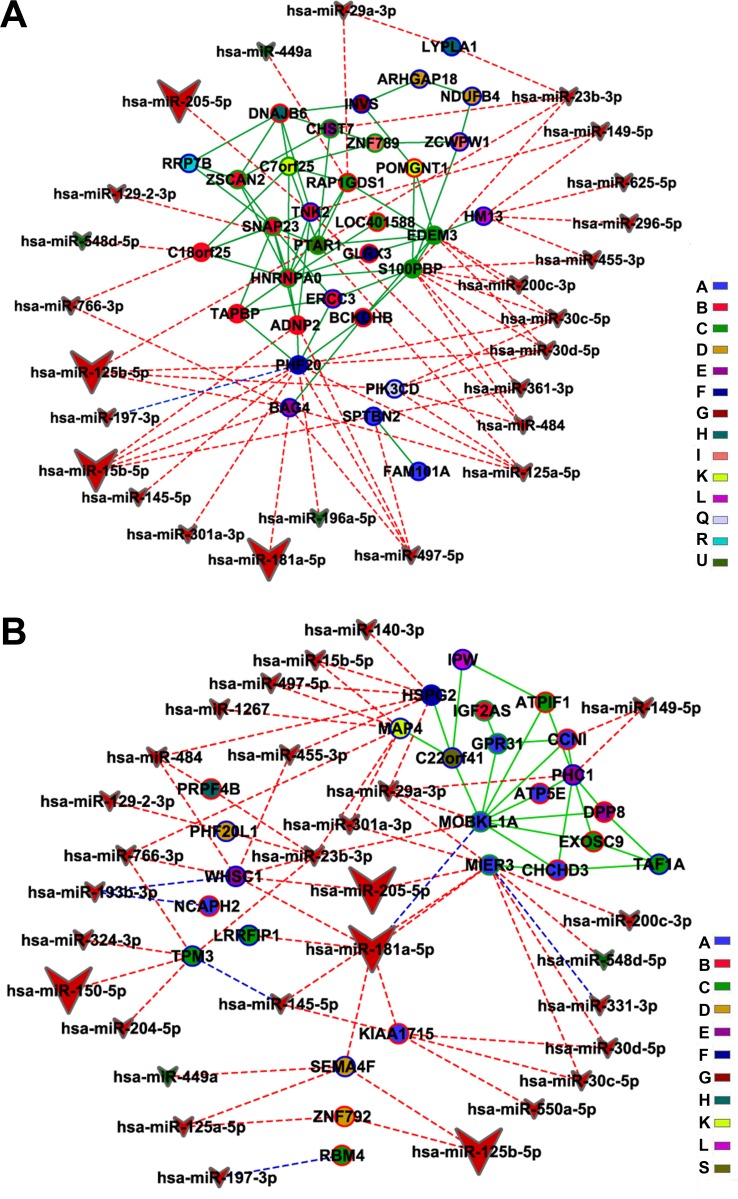
Integrative network analysis for CO subnetworks-miRNA High-hierarchy gene-miRNA interactions for CT-CO (**A**) and DS-CO (**B**) groups are depicted. Hubs, VIPs and High-hubs are indicated respectively by blue, red or green colored node borders. Green line links indicate gene-gene interactions and blue or red doted links indicate respectively validated or predicted miRNA-gene interactions. Different node colors correspond to different communities.

All CT- and DS-CO communities harboring one or more HH genes are considered in this section. As it was previously done for DE networks, community characterization includes a brief functional description of HH genes and their interaction with differentially expressed miRNAs (Tables 3 and 4). Integrative networks for differentially expressed miRNAs and CT-CO and DS-CO high-hierarchy genes appear, respectively, in Figure 8A and 8B, and will be commented in further sections.

### CT-CO network

CT-CO network harbors 14 communities containing HH genes (Table 3). Three of these communities, namely B, C and F, contain most of the network's HH genes (15 out of 32). The relevance of these communities for network functioning is evidenced in the CGCS diagram presented in Figure 4C and the biological functions of their HH genes are described just below.

Community B encompasses eight HH genes which can be divided in two subsets based on their biological functions: the larger subset encompass six HH genes related to thymocyte development whereas the smaller subset contains two HH genes acting on transcriptional regulation.

The larger subset in community B has only one high-hub, *SNAP23*, which stabilizes SNARE complexes orchestrating ERC-phagosome fusion and enrichment of phagosomes with ERC-derived MHC-I [145]. Moreover, SNARE protein VAMP8 interacts with SNAP23 and has a specific function in the thymic stroma affecting the proliferation and apoptosis of T-lymphocytes during maturation in the thymus [146].

There are three VIPs in the larger subset of community B. One is *ADNP2*, which codes for the Activity-dependent Neuroprotective Protein (ADNP) and is probably involved in protecting developing thymocytes from apoptosis, being regulated by the vasoactive intestinal peptide [147, 148, 149, 150]. The other VIP is *C18orf25* (aliase *ARKL1*), whose gene product binds CK2β [151], a component of the CD5-CK2 activation pathway that sets the threshold for thymocyte progression to double-positive stage [152]. The third VIP is *TAPBP*, coding for tapasin, a transmembrane glycoprotein which mediates interaction between newly assembled major histocompatibility complex (MHC) class I molecules and the transporter associated with antigen processing (TAP) [153].

The two remaining genes in this larger subset are hubs. One is *ERCC3* (aliase *XPB*), a gene with increased expression in Down syndrome [154] which codes for an essential core subunit of the eukaryotic basal transcription factor complex TFIIH [155]: defective TFIIH results, via TAF7, in thymocyte failure to reach DN4 stage [156]. The other hub is *TNK2* (aliases *ACK*, *ACK1*), coding for Cdc42-associated kinase I, a component of EGF receptor signaling complex that regulates EGF receptor degradation [157], thus enhancing the ability of thymic epithelial cells to sustain thymocyte differentiation [158].

The smaller subset in community B is related to transcriptional control and contains two high-hubs: *ZSCAN2*, which codes for a C2H2 zinc finger protein involved in transcriptional regulation [159], and *HNRNPA0*, a RNA binding protein that regulates transcript stability via binding to AU-rich elements of mRNA [160].

Community C harbors four HH genes (Table 3), and three of these genes are associated to thymocyte maturation processes and thymus development. In fact, *EDEM3*, a high- hub, has a relevant role in the degradation of misfolded MHC-I, MHC-II and invariant chains in the ER, critical events for antigen presentation in the thymic environment [161, 162, 163]. The other high-hub in this community is *S100PBP*, a gene coding for the binding-partner of S100P, a member of the S100 family of proteins [164]. S100P mediates cell migration and is very similar to S100B: both proteins are interactors of RAGE [164, 165], i.e. the receptor of advanced glycan end-products. RAGE is expressed in thymocytes, thymic macrophages, thymic medulla and Hassal's corpuscles and influences thymic morphology and functions [166], The third gene, a VIP, is *RAP1GDS1*, a regulator of thymocyte apoptosis via transglutaminase 2 [167]. The remaining HH gene in this community is a VIP, *LOC401588* (aliase *ZNF674-AS1*), which codes for an antisense long non-coding RNA of hitherto unknown function.

Community F harbors three HH genes (Table 3), two of them exerting relevant roles in the thymus and in the immune system. The hub, *PHF20*, codes for the plant homeodomain protein 20 which regulates NF-κB activation [168]. Here is sufficient to say that noncanonical NF-κB signaling has a significant contribution to central tolerance (and to peripheral tolerance as well) and, consequently, to thymus function [reviewed in 169]. *GLRX3* is a VIP and codes for glutaredoxin 3, or PICOT, a regulator of calcineurin-NFAT signaling [170]. Calcineurin-NFAT signaling sets the bandwidths for selecting signals during thymocyte development [107]. The other VIP is *BCKDHB* which codes for a mitochondrial protein involved the catabolism of branched-chain amino acids (BCAA). Although BCCA exert widespread effects on the immune system [171], their role in thymus awaits further investigations.

The remaining 11 CT-CO communities harbor only one or two HH genes However, some of these communities have high connection weights (Figure 5) and their HH genes exert relevant roles in the thymus, as described below.

Considering the above profile, community A is quite an exception: it spite of having the largest number of genes (404) in CT-CO network (Figure 4C, Table S2 ), it has the lowest connection weights (Figure 5) and only two HH genes, both categorized as hubs: *SPTBN2* (aliase *SCA5*), which codes for β-III spectrin, a protein necessary for endoplasmic reticulum-to-Golgi and post-Golgi protein transport [172], and *FAM101A*, which codes for a protein (family with sequence similarity 101, member A) with a hitherto unknown biological function.

The following five communities - ordered according to the number of genes harbored, from higher to lower (Table S2) - are D, E, G, H, and I. Three of these communities, D, E and H, include HH genes playing relevant roles in thymus functioning and will be commented first.

Community D has two hubs. One is *ARHGAP18*, a regulator of RhoA which interacts with moesin [173] and is involved in T-cell egress from thymus [174]. The other is *NDUFB4*, which codes for a non-catalytic subunit of the multisubunit NADH:ubiquinone oxidoreductase, the first enzyme complex in the mitochondrial electron transport chain (complex I) [175]. Besides its role in mitochondrial biology, no specific thymic function was assigned to *NDUFB4* so far.

Community E has one hub, *BAG4*, also known as *SODD*, which is critical for the regulation of TNF signaling [176, 177]. TNF regulates thymocyte production [178] and interferes with thymic emigration (thymic output) [179]. The other HH gene in this community is *CHST7*, a high-hub, whose encoded protein catalyzes the sulfation of 6-hydroxyl group of GalNAc in chondroitin. Chondroitin is secreted in large amounts by thymic nurse cells (TNCs) [180]. TNCs provide the microenvironment for secondary T-cell receptor α rearrangement in cortical thymocytes [181].

Community H also has two HH genes. One is a VIP, *DNAJB6*, whose gene product, an Hsp40 family chaperone, is able to enhance the expression of Schlafen 1 [182], a protein expressed in the thymus which regulates thymocyte development [183]. The other is *LYPLA1*, a hub which codes for APT1, an alpha/beta hydrolase involved in Ras localization and signaling [184]. Ras signaling is critical for setting thymic selection thresholds [185].

Community G has only one hub, *INVS*, which codes for the ciliary protein inversin, involved in cell polarity and migration [186], but whose function in thymus remains unknown. Community I is the last in this set and has a rather low connection weight (Figure 5). This community contains just one hub, *ZCWPW1*, and a high-hub, *ZNF789*, both coding for zinc finger proteins. ZCWPW was characterized as a histone modification reader [187] and ZNF789 (HGNC 27801) belongs to the C2H2 zinc finger domain family of transcriptional regulatory proteins.

Finally, and also listed according to the number of nodes/genes in a decreasing order (Table S2), are communities K, L, Q, R and U. Community K include two HH genes. One is *POMGNT1*, a VIP, which codes for an enzyme that participates in the glycosylation of dystroglycan [188], a crucial step for thymocyte development in mice [189]. The other HH gene community K is the high-hub *C7orf25* (aliase *MRLP32*), encoding for the mitochondrial ribosome protein L32 (protein synthesis in the mitochondrion). Each of the following four communities harbors a single HH gene. Community L contains the hub *HM13* (aliase *SPP*) which encodes a signal peptide peptidase involved in MHC-I presentation [190]. In community Q there is a hub, *PIK3CD*, which codes for class I phosphoinositide-3-kinase delta, a member of the PI3Ks molecule family whose function is to act in concert to protect thymocytes from apoptosis [191]. Community R harbors another hub, *RRP7B*, a ribosomal RNA processing gene with a yet unknown function. Community U, the last in this sequence, has a high-hub, *PTAR1,* which codes for a prenyltransferase alpha subunit. CD43, an abundant thymocyte cell surface glycoprotein acting on thymocyte development and T-cell activation processes [192], depends on post-translational prenylation for a correct subcellular localization and membrane anchoring [193].

The overall scenario of CT-CO network shows that the communities B, C and F - which contains almost half of the network's HH genes and have relatively high connection weights (Figures 4C and 5C) - are mostly related to relevant thymic pathways, such as thymocyte development and selection. A similar functional profile was found for communities D, E and for community H, which has the highest connection weight in the CT-CO (Figure 5C). Conversely, the other CT-CO communities harbor HH genes playing a rather ancillary role in support of thymic selection pathways. Hence, CT-CO gene communities would well represent a “canonical” picture of thymus functioning.

### MicroRNA target interactions in CT-CO

In CT-CO network nine out of 14 communities have genes interacting with differentially expressed miRNAs. In six of these communities there are interactions with miRNAs that are abundantly expressed in thymic tissue (Table 3 and Figure 8A). The main targets of these abundantly expressed miRNAs are genes related to thymocyte development. These genes are harbored in communities B, F, E and Q (Table 3). In communities F and E, the HH genes involved in such interactions - *PHFL20* and *BAG4*, respectively - are related to the regulation of TNF/NF-κB signaling, which is critical for relevant thymic pathways, like central tolerance and thymocyte development [169]. These two genes have the highest numbers of miRNA interactions, as well as interactions with abundantly expressed miRNAs, in CT-CO network (Table 3 and Figure 8A), what indicates that their expression is tightly buffered [94]. A similar situation occurs in community Q, where the sole HH gene, *PIK3CD* - a hub related to thymocyte protection from apoptosis - is under tight miRNA control. In community B two genes are targets of abundantly expressed miRNAs - *ADNP2* and *TNK2* - both related to stromal cells and thymocyte development. Therefore, the genes related to TNF signaling and thymocyte development are those with more miRNA interactions in CT-CO, including abundantly expressed miRNAs.

The other CT-CO communities present lower numbers of miRNA interactions. The HH genes involved in these interactions are associated to cellular and metabolic processes necessary for thymic functioning (Golgi transport, prenylation, Ras signaling), or in MHC antigen presentation. The only exception is community C: it has more miRNA interactions than any other community in CT-CO, but none of these interactions are with abundantly expressed miRNAs. All the three HH genes coding for proteins in this community (the fourth gene is a long non-coding RNA) have miRNA interactions and exert diverse and relevant thymic functions in thymus, as described in the previous section.

### DS-CO network

The DS-CO network has 12 communities containing HH genes. The majority of these genes (20 out 31) belong to four communities - A, C, D and E - which also concentrate most of the genes with higher numbers of miRNA-gene interactions (Table 4). The relationship among DS-CO communities is depicted in the CGCS diagram presented in Figure 4D. The biological functions of the HH genes present in all these communities are summarized below.

Community A is the largest DS-CO community (Table S2) both in number of genes (587) and of HH genes (11). All HH genes in this community are VIPs or high-hubs, therefore playing significant roles in network's functionality and robustness [24, 25]. Moreover, these HH genes form three distinct functional subsets, as commented below.

The first HH gene subset to be considered in this community - formed by two high-hubs and two VIPs - is mostly related to T-cell development. One of these high-hubs is *GPR31*, which codes for G protein-coupled receptor 3, a member of GPCR superfamily involved in the activation of ERK1/2, MEK, and NF-κB pathways [194, 84] and, therefore, in T-cell development processes [195, 196]. The other high-hub is *MOBKL1A* (aliase *MOB1B*), which codes for a kinase regulator [197]. Mst1/2-catalyzed *MOB1B* phosphorylation controls, via Dock8, the migratory responses of single positive thymocytes [38]. One of the VIPs, *CCNI*, codes for cyclin I, a cyclin family member that controls cell cycle progression by regulating cyclin-dependent kinases [198]. Cyclin I belongs to Wnt/beta-catenin signaling pathway, which regulates the late stages (positive selection) of thymocyte development [199]. The other VIP, *NCAPH2*, codes for the kleisin-β subunit (CAP-H2) of the condensin II, which is crucial for the developmental progression of DN4 thymocytes [200].

The second subset of HH genes in community A is constituted by four VIPs associated with thymic metabolic processes. Two of these genes are mitochondrial related: *ATP5E*, codes for the subunit epsilon of mitochondrial ATP synthase, a key enzyme of mitochondrial energy provision [201], and *CHCHD3* codifies for an inner mitochondrial membrane protein, essential for maintaining crista integrity and mitochondrial function [202]. The other two VIPs are: *KIAA1715* (aliases *LNP*, *LNP1*) a member of Lunapark family which plays a key role in ER tubular network organization [203], and *TMOD4* that encodes tropomodulin 4, whose expression is decreased in myogenesis while increased in adipogenesis [204]. It is known that along thymic demise there is a progressive replacement of lymphostromal thymic zones with adipocytes [205], what is also clearly observed in the thymuses of DS infants (see Figure S2).

The third HH gene subset in community A is constituted by three high-hubs; all related to epigenetic or transcriptional control mechanisms. Accordingly, *MIER3* codifies for a mesoderm induction early response 1, family member 3, which is an ELM-SANT domain protein and interacts with HDAC1 [206]. The other two HH genes - *ZMYM1* and *ZNF625* - codify for zinc finger proteins. *ZNF625* belongs to the C2H2 family of zinc finger proteins, which act as trans-regulators of gene expression [207]. *ZMYM1* belongs to the MYM-type zinc finger family. Although most members of this family are involved in transcriptional control [208], the specific functional role of the ZMYM1 protein is yet undetermined.

Community C encompasses three VIPs and three hubs and four of these genes are involved in RNA metabolism and transcriptional control. The VIPs are linked to basic cellular and molecular processes in thymus: *ATPIF1* codifies a mitochondrial ATPase inhibitor involved in mitophagy [209], an essential process for the development of thymic iNKT cells in mice and humans [69]; *EXOSC9* (aliase *RRP45*) is involved in RNA turnover and quality control, encoding an exoribonuclease complex which degrades mRNAs containing ARE (AU-rich elements) [210]; and *RBM4* (aliase *LARK*) codifies a RNA-binding protein involved in alternative splicing of mRNA [211] and in miRNA-guided gene regulation [211, 212]. Interestingly, RBM4 is significantly decreased in the fetal brain in Down syndrome [213]. Two of the three hubs in community C also play significant roles in thymus functioning. *LRRFIP1*, one of these hubs, is a transcriptional repressor and acts with noncoding RNAs to control TNF expression [214]. Elevated TNF expression leads to thymic atrophy [215]. Another hub, *TPM3*, codes for tropomyosin 3, an actin binding protein involved in actin dynamics and cell migration [216, 217]. As it will be commented in the following section, this gene has many miRNA interactions (validated and predicted) in the thymic environment. The last hub in this community, *TAF1A*, codes for a TATA-box binding protein and regulates transcription initiation [218].

Community D has two hubs and one VIP. One hub is *PHF20L1*, which stabilizes DNA (cytosine-5) methyltransferase 1 (DMNT1) and regulates DNA methylation in cells. This gene is an epigenetic reader (methyllysine reader) and cooperates with writer and eraser to regulate epigenetic inheritance [219]. The other hub is *SEMA4F*, which codes for a transmembrane class IV semaphorin (semaphorin 4A) dynamically regulated along thymocyte development [220]. Semaphorins exerts a role on T-cell migration [221] and are miRNA regulated [222]. The VIP is *ZNF792*, which codes for a C2H2-type zinc finger protein (HGNC 24751), possibly involved in gene expression regulation, but whose specific function in the thymus is as yet unknown.

Community E has three HH genes: one VIP and one hub are associated to thymic T-cell development, and the other hub acts on epigenetic control mechanisms. *DDP8*, a VIP, codes for dipeptidyl peptidase 8. This protein is structurally and functionally very similar to dipeptidyl peptidase IV (CD26) [223; 224] and probably exerts the same role as CD26 does in maintaining thymic architecture and thymocyte proliferation during immunosenescence [225]. The hub *PHC1* (aliase *RAE28*) codes for a Polycomb gene involved in the repression of HOX genes [226] and the knocking out of this gene in mice causes thymic T-cell arrest at CD4^−^ CD8^−^ double-negative immature stage [227]. The second hub, *WHSC1* (aliases *MMSET*, *NS2*) codes for a histone methyltransferase and is a chromatin modifier controlled by a network of miRNAs [228].

Each of the other eight communities in DS-CO network harbors just one HH gene. However, only in three of these communities - F, H and K - the HH genes were found to have interactions with several miRNAs (Table 4). The relatively high number of such interactions indicates that their transcriptional programs are under a somewhat more robust control in network conditions [94]. One of these genes is the hub *HSPG2*, in community F, which codes for the protein pelercan, an integrant of the lamimin-5 contaning conduits in human thymus. The conduit system is present in the medulla of human thymus and it is responsible for the transport of small blood-born molecules to defined sites within the medulla [229]. In community H there is the VIP, *PRPF4B* (aliase *PPR4*), which codes for a serine/threonine protein kinase that binds the Krüppel-like factor 13 (KLF13) in human thymocytes and promotes thymocyte survival [230]. Lastly, in community K there is the hub *MAP4*, which encodes a microtubule binding protein. MAP4 is microtubule-associate and growth regulator protein [231] and interacts with Septin 9 (Sept9) [232] in order to modulate microtubule dynamics. Sept9, a filament-forming protein, exerts a crucial role in the transition from the double-negative stage during thymocyte development [233]. The remaining five HH genes which do not have assigned miRNA interactions in DS-CO network are described below.

The two single hubs in communities B and L (Table 4) are paternally imprinted genes: *IGF2AS*, in community B, encodes a long non-coding RNA (lncRNA), the antisense transcript of the insulin-growth factor 2 gene [234], and *IPW*, in community L, codes a lncRNA widely expressed in adult and fetal tissues and associated to Prader-Willi syndrome [235, 236]. The lncRNAs are usually involved in transcriptional control and epigenetic mechanisms, but the specific functions of *IGF2AS* and *IPW* in the thymus are presently unknown. The hub in community G, *RPS2*, codes for a highly conserved 40S subunit ribosomal protein [237]. RPS2 is known to be overexpressed in some human tumors [238], but a specific role for this protein in thymus has not yet been described. Community S has the hub *C22orf41* (aliase *SYCE3*), which codes for the synaptonemal complex central element protein 3 [239], with unknown functions in thymic tissue. Finally, community Y harbors as its sole HH gene the hub *ST13*. This gene codes for the Hsc70-interacting protein (Hip). Hip promotes the functional maturation of the glucocorticoid receptor (GR) [240]. GR is constitutively expressed in thymocytes and plays a significant role in thymic homeostasis [241, 242], mediating physiological and stress responses [242, 243, 244].

As a whole, the functional profile of HH genes in DS-CO communities differs from that found for CT-CO network. Firstly, the DS-CO network contains a sizable number of HH genes related to epigenetic mechanisms (seven genes) and transcriptional control (five genes). Altogether, 12 out of 31 HH genes in DS-CO have these functions, against only 5 (all related to transcriptional control) out of 32 in CT-CO. Secondly: in DS-CO network there is a comparatively low number of HH genes directly involved in thymic selection pathways: just seven against 13 in CT-CO. Interestingly, DS-CO has three HH genes associated to cell migration processes that present several miRNA interactions (see below), and two HH genes associated to thymic demise (*TMOD4* and *DPP8*). Altogether, the DS-CO network depicts a rather “non-canonical” way of thymus functioning, probably enforced by epigenetic mechanisms. This issue will be further detailed in the Discussion session. The pie chart depicted in Figure 10 shows a compative view of HH gene functional profiles in CT-CO (Figure 10A) and DS-CO (Figure 10B) network.

**Figure 9:**
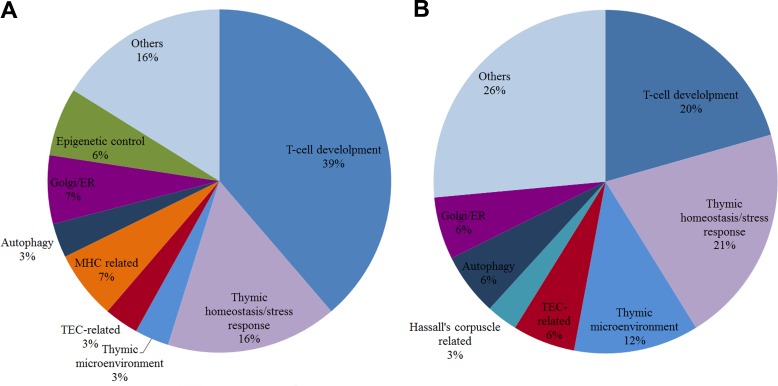
Pie chart of biological functions for high-hierarchy genes in DE networks Pie chart of HH gene functions in CT-DE network (**A**) and DS-DE network (**B**). Each slice represents the percentage of genes in a functional category.

**Figure 10:**
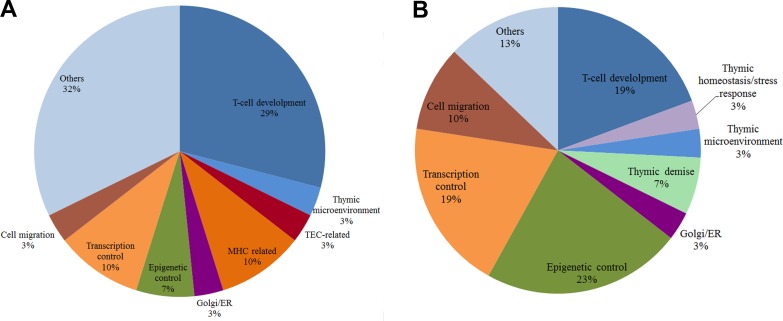
Pie chart of biological functions for high-hierarchy genes in CO networks Pie chart of HH gene functions in CT-CO network (**A)** and DS-CO network (**B)**. Each slice represents the percentage of genes in a functional category.

### MicroRNA target interactions in DS-CO

In DS-CO network most of the interactions with abundantly expressed miRNAs, as well a significant part of all gene-miRNA interactions, occur with HH genes related to epigenetic processes, cell migration and transcription control (Table 4 and Figure 8B). These genes are concentrated in communities A, C, D and E. The high-hub *MIER3* in community A is related to epigenetic mechanisms and has the highest number of miRNA interactions in DS-CO (ten, including two abundantly expressed miRNAs). Other genes related to epigenetic mechanisms, like *WHSC1* in community E, show a similar “tight control”, meaning that the epigenetic processes are very robust in DS-CO. The same considerations apply for HH genes involved in cell migration processes: for instance, *TPM3* in community C and *SEMA4F* in community D. Some genes related to transcriptional control, like *LRRFIP1* in community C and *ZNF792* in community D, have interaction with abundantly expressed miRNAs. This general picture confirms the importance and robustness of epigenetic, cell migration and transcriptional control processes, and could be considered the functional signature or DS-CO. The two other genes with relevant miRNA interactions in DS-CO are *HSPG2*, in community F, and *MAP4* in community K. The first codes for pelercan, an integrant of the conduit system for small molecules in thymic medullar area, and the former codes for a microtubule protein involved in thymocyte development.

### Interactome network analysis

Only the genes categorized as hubs, VIPs or high-hubs were considered in this analysis. MINT and IntAct databases were selected for data generation, which resulted in interactomes with 355 nodes and 1,305 edges for CT-DE; 168 nodes and 396 edges for DS-DE; 139 nodes and 313 edges for CT-CO and 161 nodes and 413 edges for DS-CO.

Network visualization and analysis were obtaining after filtering for HH gene products, excepting the ubiquitously distributed proteins (HSPA1A, APP and RNF11 for CT-DE; CASP4 and PTPN12 for DS-DE; SNAP23, TNK2, GLRX3, HNRNPA0 and DNAJB6 for CT-CO; and ST13, MAP4, TPM3 and PRPF4B for SD-CO). These networks include nodes at the first level and all links connecting these nodes, resulting in interactomes with 151 nodes and 319 edges for CT-DE (Figure S3A); 128 nodes and 252 edges for DS-DE (Figure S3B); 76 nodes and 111 edges for CT-CO (Figure S3C) and 98 nodes and 205 edges for DS-CO (Figure S3D). A functional description of the HH gene products and their interacting protein (first level) based on Gene Ontology (biological process) and PubMed databases is presented in Tables S4-S7 for DE and CO networks.

The interactome analysis shows, for all networks, a reasonable coherence among gene-gene and protein-protein interaction data, namely, the functional profiles obtained from GCNs and interactome networks are quite similar. In fact, Tables S4-S7 and Figures S3 A-D show a functional association between GCN communities and the first neighbor protein clusters.

### qPCR validation

In order to technically validate the DNA microarray data five up-regulated genes in DS group - *DEFA3*, *DEFA4*, *LFT*, *S100P* and *TMEM45A* - were selected for real-time quantitative PCR (qPCR) analysis. The fold-changes for each gene, comparing DS versus CT group's average relative gene expression, confirmed DNA microarray gene expression results (Figure S4).

## DISCUSSION

The impact of trisomy 21 on thymic gene interaction networks was assessed by means of comparative genomic and topological analyses of GCNs - obtained for differential expressed genes and for the global gene expression - in thymic tissues of DS and karyotipically normal subjects. We were able to show that thymic genomic dysregulation in Down syndrome is characterized by distinctive features regarding GCN topology and node hierarchy, derived from widespread changes in the transcriptional program of thymic cells. These data were integrated with miRNA target analysis in order to investigate the mechanism by which trisomy 21 alters the transcriptional program in the thymus of DS subjects.

The trisomy 21 impact in DE networks is clearly evidenced by the pronounced differences in network topology between CT and DS groups (Figure 3A and 3B). The high modularity of DS-DE network contrasts with its reduced connectivity, thus reflecting the disorganization of modular interactions caused by gene dosage imbalance [11, 29, 245]. The CGCS diagrams (Figure 4A and 4B) and the connection weight values obtained for all the communities in CT-DE and DS-DE networks (Figure 5A and 5B) corroborate this picture. The functional profile of HH genes in CT-DE (Table 1) shows that more than one third of these genes are related to T-cell development (12 out of 32), while this functional category encompasses only one fifth of the HH genes in DS-DE (7 out of 34). Conversely, in DS-DE eleven HH genes are involved in thymic homeostasis/microenvironment and stress tolerance processes, whereas in CT-DE these categories sum up only 7 genes. In both DE networks the genes belonging to these most prominent functional categories are targeted by abundantly expressed miRNAs (Tables 1 and 2, Figures 6 and 7).

The scenario portrayed above show that HH genes in CT-DE network are mostly associated with the main thymic pathways, such as T-cell development, whereas in DS-DE part of the HH genes serve to keep up a rather different transcriptional program, where stress tolerance and thymic homeostasis gain relevance. This is quite understandable considering that oxidative damage is an early event (detected in DS pregnancy) in the DS pathogenesis and might contribute to the development of deleterious DS phenotypes [246]. In fact, individuals with DS have high levels of oxidative stress throughout the lifespan [247]. Moreover, thymic stromal cells normally express the amyloid precursor protein, APP [62]. The overexpression of APP, a hallmark of Down syndrome [87], is known to be associated with oxidative stress [248]. Therefore DS thymus development takes place under oxidative stress conditions, what is in agreement with imaging data showing that DS thymic involution starts in utero [16]. The comparative analysis of DE gene networks clearly shows a genomic adaptation of DS-DE network in order to cope with a stressing environment. The transition mechanisms underlying such adaptation will be discussed further in this section.

In the CO network the adaptive mechanisms commented above are yet more evident. CT-CO network has 14 communities containing HH genes out of a total of 27 communities (Table S2 and Table 3). However, just three of these 14 communities - B, C and F, with relatively high connection weights - encompass half of the network's HH genes and these genes are mostly related to relevant thymic pathways, such as thymocyte development and selection (Table 3, Figures 4C and 5C). These three communities and communities E, Q and U, also harboring only genes directly related to T-cell development, happen to contain almost all the targets of abundantly expressed miRNAs in CT-CO network (Table 3, Figure 8A). Therefore, as stated before, CT-CO HH genes and communities seem to be engaged in a “canonical” way of thymus functioning and the transcriptional program behind such way is robustly buffered by miRNA interactions.

The DS-CO network, on the other hand, presents a rather different functional picture. In this network almost half of its 32 HH genes take part in epigenetic and transcriptional control mechanisms (Table 4). These HH genes (and their communities) also contribute with most of the interactions with abundantly expressed miRNA in DS-CO network. The HH genes involved in T-cell development are just seven and only one, *MAP4*, interacts with an abundantly expressed miRNA. Hence, in this network the transcriptional program is biased towards a rather “non-canonical” way of thymus functioning, probably enforced by epigenetic mechanisms acting at the chromatin level and involving miRNAs as well. Interestingly, DS-CO has two HH genes associated to thymic demise processes, as mentioned before. As a whole, this genomic profile possibly represent an adaptation to precocious thymic involution in DS, where functional and histological alterations occur, such as the cortical atrophy observed in DS subjects included in this study (Figure S2).

In summary, the genomic changes induced by trisomy 21 in the thymic tissue may well be depicted as the breakdown and altered reorganization of transcriptional modules. In terms of network biology, this process implies in a qualitative transition in the gene network from normal state to disease state. This transition involves a transient decrease in network robustness, with loss of network connectivity and subsequent network reorganization, with changes in node hierarchy and under a robust but distinct modular architecture [249]. This kind of network transition happens in GCNs via gain or loss of edges between their constituent nodes, i.e. between their genes [26, 245]. The identification of these transitions and their leading biological networks has been considered essential for unveiling the molecular and genomic mechanisms underlying chronic diseases, such as cancer or epilepsy [26, 250], as well as in genomic-driven network perturbations, such as trisomy 21 [105, 245].

The integration of community structure (modular transcriptional repertoire) and miRNA target analyses allowed the identification of the leading gene coexpression networks (GCNs) that correspond to thymus functioning in karyotypically normal and DS subjects. Here is important to remember that DE networks are subnetworks of the global gene expression networks, or CO networks. The comparative analysis of DE networks probably portrays the “ground zero”: the subnetwork transition considering only the genes whose transcription was significantly altered by trisomy 21. On the other hand, the comparative analysis of CO networks reveals the derived “shock-waves” of trisomy 21 genomic dysregulation, reflecting its effects on the organ's global transcriptional program. This is in accordance with recent findings showing that trisomy 21 modifies the cell's transcriptional program through expression dysregulation domains dispersed along different chromosomes in the genome [11].

In conclusion, the CT networks - and principally the CT-CO network - would depict the “canonical” way of thymus functioning. Conversely, the DS networks represent a “non-canonical” way, what means the thymic tissue adaptation to trisomy 21 genomic dysregulation and it's functioning under stressed conditions. This adaptation is probably driven by epigenetic mechanisms acting at chromatin level and through the miRNA control of transcriptional programs involving the networks' high-hierarchy genes.

## MATERIALS AND METHODS

### Patients

Thymic tissue samples were obtained from 10 Down syndrome subjects and 10 karyotypically normal subjects that underwent cardiac surgery at Instituto Dante Pazzanese de Cardiologia, São Paulo, Brazil. All patients were gender- and age- matched (age ranging from two to 18 months) (Table 5, Figure S5). This research has been approved by the research ethics committee of Instituto Dante Pazzanese de Cardiologia under number 4287. A written informed consent was obtained from all patients.

**Table 5:** Clinical data

Sample ID	Gender	Age at surgery (yr/mo/d)^a^
DS1^b^	female	6mo27d
DS2^b^	male	4mo1d
DS3^b^	female	7mo24d
DS4^b^	female	7mo1d
DS5	female	1yr6mo
DS6^b^	male	5mo21d
DS7^b^	female	9mo14d
DS8^b^	male	11mo11d
DS9^b^	male	1yr6mo
DS10	male	4mo29d
CT1^b^	female	6mo10d
CT2^b^	female	1yr5mo
CT3^b^	male	9mo1d
CT4^b^	male	1yr6mo
CT5	female	6mo24d
CT6^b^	female	6mo9d
CT7	male	2mo11d
CT8^b^	male	2mo1d
CT9^b^	female	9mo26d
CT10^b^	male	3mo19d

a yr: years; mo: months; d: days

b samples also used for miRNA analysis.

### Thymic tissue specimens

Fresh corticomedullar sections of thymic tissue were obtained at surgery room from DS subjects (DS group) and karyotipically normal subjects (CT group) and were immediately preserved with RNA*later* (Qiagen cat. no. 76106, Valencia, CA). Haematoxylin and eosin (HE) histology was performed for all thymic specimens and revealed typical cortical atrophy [12] in DS samples (Figure S2). Detailed morphometric and immunohistochemical analyses of this material are beyond the scope of this paper and will be published elsewhere.

### Total RNA extraction

Thymus tissue explants (3-4 mm^3^) were homogenized with TissueRupter (Qiagen, cat. no. 9001272 Valencia, CA) and total RNA was extracted from the homogenates using the RNeasy Lipid Tissue Kit (Qiagen cat. no. 74804, Valencia, CA) according to the manufacturer's instructions. RNA quality was assessed on the Agilent BioAnalyzer 2100 (Agilent, Santa Clara, CA). All samples were stored at −80°C until used in hybridization experiments.

### Microarray hybridization and gene expression analysis

In order to determine gene expression profiles, 4x44K v.2 DNA microarrrays (Whole Human Genome Microarray Kit, Agilent Technologies, cat no. G4845A, Santa Clara, CA) were used. The procedures for hybridization using the fluorescent dye Cy3 followed the manufacturer's protocols (One-Color Microarray-Based Gene Expression Analysis - Quick Amp Labeling). The images were captured by the reader Agilent Bundle according to the parameters recommended for bioarrays and extracted by Agilent Feature Extraction software version 9.5.3. Spots with two or more flags (low intensity, saturation, controls, etc.) were considered as NA, that is, without valid expression value. The R software version 2.11.1 [251] and an in house script were used for: i) sample grouping (CT or DS groups); ii) excluding transcript spots presenting three or more NAs per group; iii) converting gene expression values to log base 2. Through this procedure we identified 12,989 valid GO annotated genes for DS and CT groups. By means of the TMEV software version 4.6.1 [252] we obtained the 538 differentially expressed (DE) GO annotated genes using the Significance Analysis of Microarrays (SAM) procedure. All microarray raw data have been deposited in GEO public database (http://www.ncbi.nlm.nih.gov/geo), a MIAME compliant database, under accession number GSE69210.

### MicroRNA microarray hybridization and analysis

Total RNA samples obtained from eight Down syndrome subjects and eight karyotypically normal subjects (Table 5) were used to determine and evaluate the miRNA profiles. Whole human miRNA of 8x15K DNA microarrays (Human miRNA Microarray Kit V3, G4470C, Agilent Technologies), containing probes for 866 human and 89 viral miRNAs based on Sanger miRBase (release 12.0) were used. The procedures for hybridization followed the protocols provided by the manufacturer's instructions (miRNA Complete Labeling and Hyb Kit, Agilent Technologies, cat. no. 5190-0456). The images were captured by the reader Agilent Bundle according to the parameters recommended for bioarrays and extracted by Agilent Feature Extraction software version 10.7.3. Spots with two or more flags (low intensity, saturation, controls, etc.) were considered as NA, that is, without valid expression value. The R software version 2.11.1 [251] and an in house script were used for: i) sample grouping (CT or DS groups); ii) excluding transcript spots presenting two or more NAs per group; iii) converting miRNA expression values to log base 2. Through this procedure we identified 641 valid miRNAs for DS and CT groups. Differentially expressed miRNAs were obtained by means of the TMEV software version 4.6.1 [252] using the unpaired t-test (p<0.05). All microarray raw data have been deposited in GEO public database (http://www.ncbi.nlm.nih.gov/geo), a MIAME compliant database, under accession number GSE70573.

### Gene coexpression networks (GCNs): visualization, analysis and community detection

Gene coexpression networks for differentially expressed GO annotated genes (DE networks) for all valid GO annotated genes, namely complete networks (CO networks), were constructed for DS and CT groups based on Pearson's correlation, as we previously described [24]. Pearson's correlation identifies sets of genes which covaries (positively or negatively), thus allowing us to construct networks by considering nodes as genes, with edges inferred if a pair presents high absolute value of correlation. Specifically, we define a correlation threshold that determines if edges are present or absent in the resulting network. This is done in a way that all nodes are connected to the major component and the network is stable in the sense that slight changes in the threshold value do not significantly affect its topological structure [24]. Networks were tested for scale free status by Kolmogorov-Smirnov (K-S) statistics, i.e. power law distributions in empirical data [253].

As these networks may grow larger in the number of components (e.g. hundreds or thousands) or present very intricate connections between them (such as hierarchical or modular structure), it becomes mandatory the use complex network analysis methodology to better characterize such networks [25, 33, 254].

We developed a network methodology for GCN visualization and analysis [24, 25, 26] that allows the categorization of network nodes according to node-centered connectivity taken along distinct hierarchical levels of gene-gene neighborhoods [255, 32]: hubs are highly connected nodes, VIPs - standing for “Very Important Person”, an acronym initially coined for the study of social networks [256] and equivalent to the term “date-hubs” in biological network papers [41] - have low node degree but connect only with hubs, and high-hubs have VIP status and high overall number of connections. We classified network nodes as VIPs, hubs or high-hubs by obtaining the node degree, *k_0_*, and the first level concentric node degree, *k_1_*, which takes into account all node connections leaving from its immediate neighborhood, then projecting all node values in a *k_0_ vs k_1_* plot. All calculations were done by using Python program and the conceptual framework is described at http://cyvision.if.sc.usp.br/~bant/hierarchical/. Along this paper, hubs, VIPs and high-hubs are sometimes designated high-hierarchy genes (HH).

### Connectivity

The network connectivity k for non-directed networks was calculated by k = 2L/N, where L stands for the number of edges and N for the number of nodes [257].

### Community detection

Community detection in complex networks is usually accomplished by discovering the network modular structure that optimizes the modularity measurement. Modularity takes into account the relationship between the number of links inside a community and between nodes in distinct communities compared to the random model [257, 258]. A diverse range of optimization techniques exist to optimize the modularity. Here we applied the method proposed by Blondel et al. [259] which attains good modularity values and presents excellent performance.

### Connection weight between communities

Connection weight values were obtained for all the constituent communities of DE networks and for all the CO networks' communities harboring high-hierarchy genes. The weight of connections *W_αβ_*, which also comprise the elements of the mixing matrix [258], is taken as the stochastic probability of a vertex in the community α connecting to a vertex in *β*, which can also be given as: *W_αβ_* = *E_αβ_*/(|*α*||*β*|), where *E_αβ_* stands for the total number of edges between the two communities *α* and *β* [260, 261]. The normalization in *W_αβ_* is needed to account for the distinct community sizes present in the network, otherwise the edges weights would become biased towards the larger communities.

### Coarse-grained community structure

As a complementary analysis for the community detection, each GCN was rearranged in a new network accounting only for the relationships between each community, also known as coarse-grained community structure (CGCS) [41, 260, 262]. Here the CGCS was generated by contracting all nodes inside each community into a single community node; likewise, edges are summarized in terms of the connection weight between such communities, *W_αβ_*. This structure can also be obtained directly by considering the mixing matrix [258] as an adjacency matrix of the new network, which has been used to summarize the community organization of many knowledge networks such as citation networks [260, 262].

### Structural analysis of the CO communities' subnetworks

Complete sets of gene expression values were obtained from each one of the CO network communities (CT and DS networks) were used for generating their corresponding subnetworks using Pearson's correlation and the same link strength threshold adopted for CO networks. Only communities harboring 100 or more genes/nodes were considered in this analysis [257]. This approach allowed the characterization of subnetworks' topology and connectivity.

### MicroRNA target analyses

Analyses of the differentially expressed miRNAs interactions with all selected high-hierarchy genes from DE and CO networks were performed based on the following miRNAs databases: miRTarBase, an experimentally validated miRNA-target gene interaction database, and mirPath database (predicted miRNA-target gene interaction). The miRNA-gene interaction networks and integrative networks (high-hierarchy gene coexpression subnetworks-miRNA) were visualized through Cytoscape 3D.

### Interactome analysis

The interactome networks were constructed using an in house free web tool developed by Leandro de A. Lima and Renato D. Puga from Centro Internacional de Pesquisa e Ensino (CIPE) - A. C. Camargo Cancer Center (http://bioinfo.lbhc.hcancer.org.br/cgi-bin/interactomegraph/index.cgi). Only categorized hubs, VIPs and high hubs genes were considered in this analysis. MINT and IntAct databases were selected for comparison and data generation. Data analysis and visualization were accomplished through Cytoscape (version 3.1.0, www.cytoscape.org).

### qPCR for microarray technical validation

Differential gene expression data were validated through quantitative real-time polymerase chain reaction (qPCR). Specific primers for five selected genes (Table S8) were designed using the Primer-BLAST (Primer3 Input, version 0.4.0 and BLAST, available at http://www.ncbi.nlm.nih.gov/tools/primer-blast/). All samples were amplified in triplicates. Amplification reactions were performed in a 25 uL final volume containing 1X SYBR Green mix (Quantitec SYBR Green PCR kit, QIAGEN, Hilden, DE), 10 pmol of each primer and 2 μL cDNA (1/10 dilution, synthesized from 1 μg of total RNA). Real time PCR amplifications were performed in Applied Biosystems StepOne Plus Real Time PCR System with StepOne software (Applied Biosystems, Foster City, CA, USA) with the following cycling parameters: an initial hot start of 95°C for 15 min followed by 50 cycles of 95°C for 15 s and 60°C for 30 s. In order to normalize qPCR reactions, *GAPDH* was included as reference gene after checking that amplification curves for 10 different RNA samples (5 from DS and 5 from CT) yielded essentially the same results. Relative expression was determined by the relative standard curve method [263] and presented as fold change comparing DS versus CT mean values.

## SUPPLEMENTARY MATERIAL FIGURES AND TABLES












