# Supplementary material for: Modular transcriptional repertoire and MicroRNA target analyses characterize genomic dysregulation in the thymus of Down syndrome infants
Source: Oncotarget. 2016 Feb 1;7(7):7497–533. doi: 10.18632/oncotarget.7120 (PMC4884935; doi:10.18632/oncotarget.7120)
Supplement: Supplementary file 1 [file oncotarget-07-7497-s001.pdf]

Modular transcriptional repertoire and MicroRNA target analyses characterize genomic dysregulation in the thymus of down syndrome infants

Supplementary Material

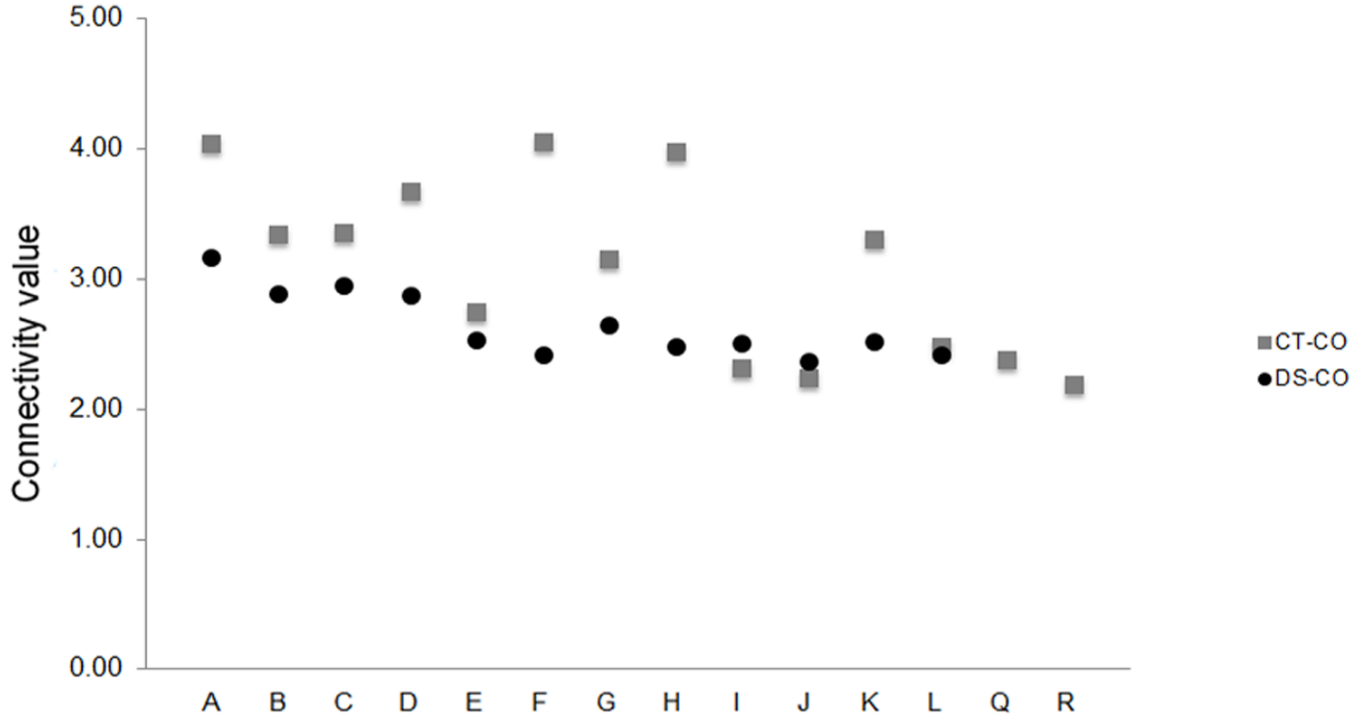

Figure S1. Community subnetwork connectivities in CO networks. Dispersion graph of connectivity values for communities' subnetworks in CT-CO (square dots) and DS-CO (circle dots) networks.

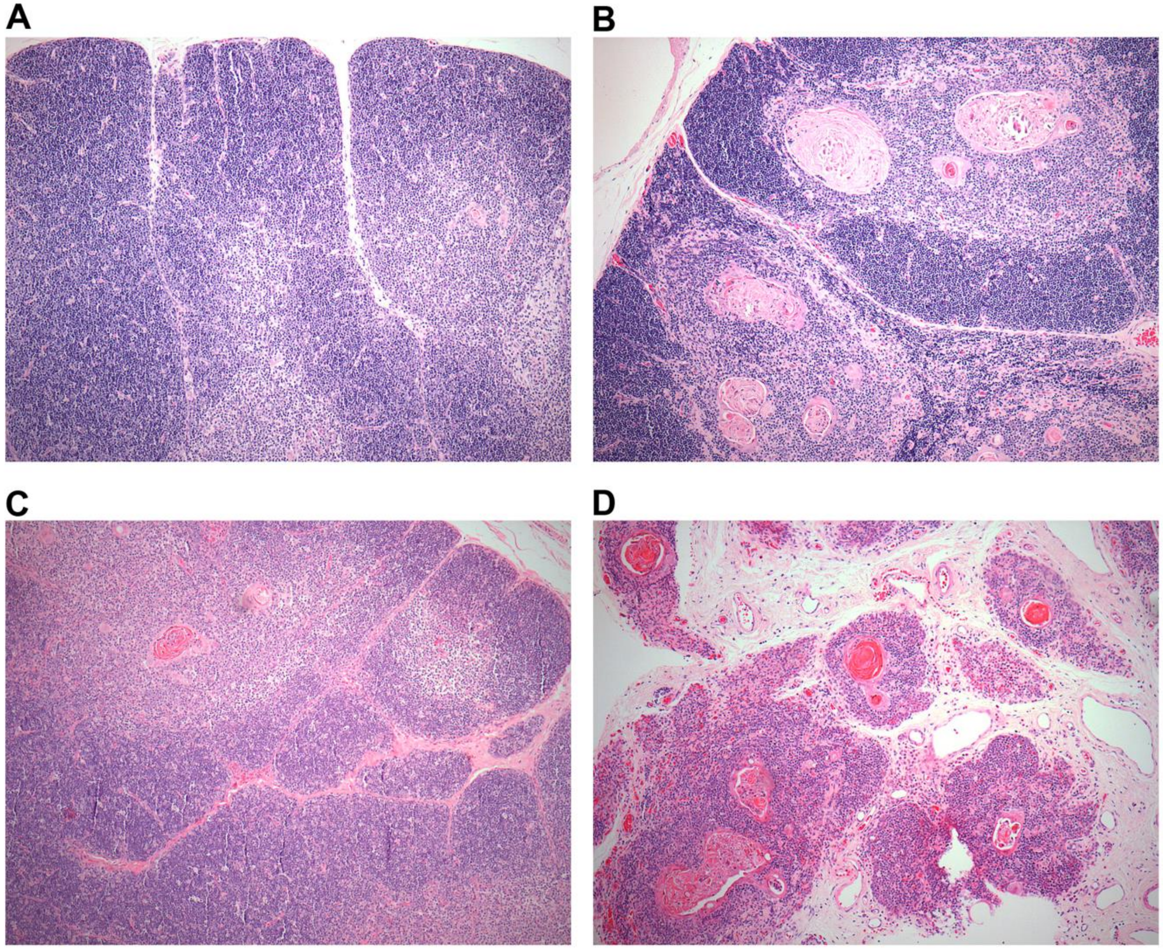

Figure S2. Histology of the Thymus. Histological aspect of thymuses obtained from karyotipically normal (**A** and **C**) and Down syndrome patients (**B** and **D**) included in this study. In **A** and **B** the subjects were 9 months old (patients CT9 and DS7). In **C** and **D** the subjects were 18 months old (patients CT4 and DS5). Cortical atrophy is present in Down syndrome thymuses. The 18 months old DS patient had more evident morphological changes, with marked cortical atrophy. All figures HE, 100X.

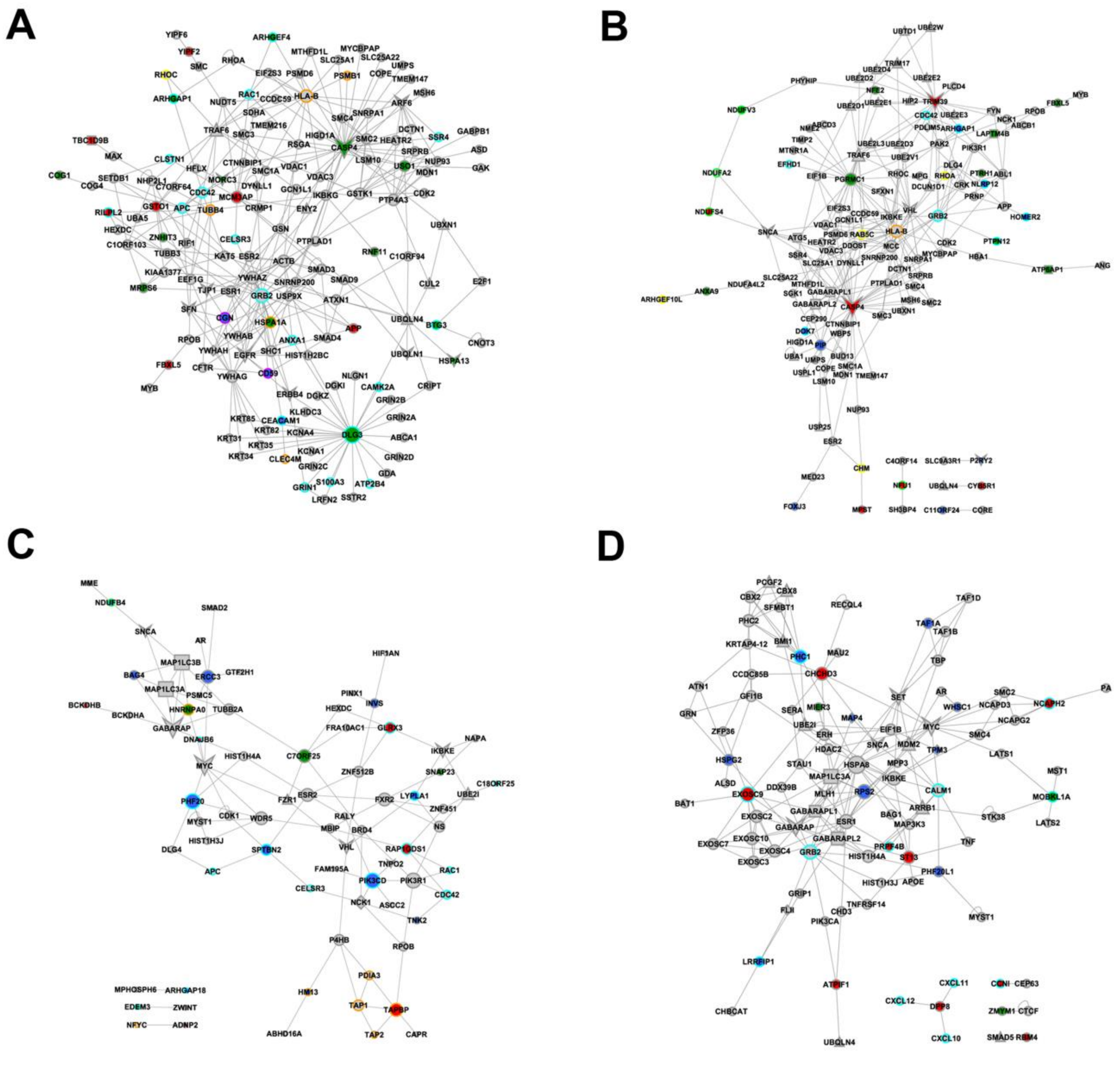
A
B
C
D

Figure S3. Interactome for DE and CO networks. Interactome for CT-DE (**A**), DS-DE (**B**), CT-CO (**C**) and DS-CO (**D**) selected hubs (depicted in blue), VIPs (depicted in red) and high-hubs (depicted in green) using MINT and IntAct databases. Node size is related to node degree (number of links). Node shapes and border colors represent biological processes, as follows: circle for cell process; circle with light blue border for calcium ion binding, thymic or T cell development and T cell differentiation, circle with orange, purple, yellow or light green border stand for, antigen presentation, Hassall´s corpuscles, signaling/GTPase activity or mitochondrial respiratory chain respectively; diamond for actin/cytoskeleton; square for autophagy; triangle for ubiquitination; vee for apoptosis.

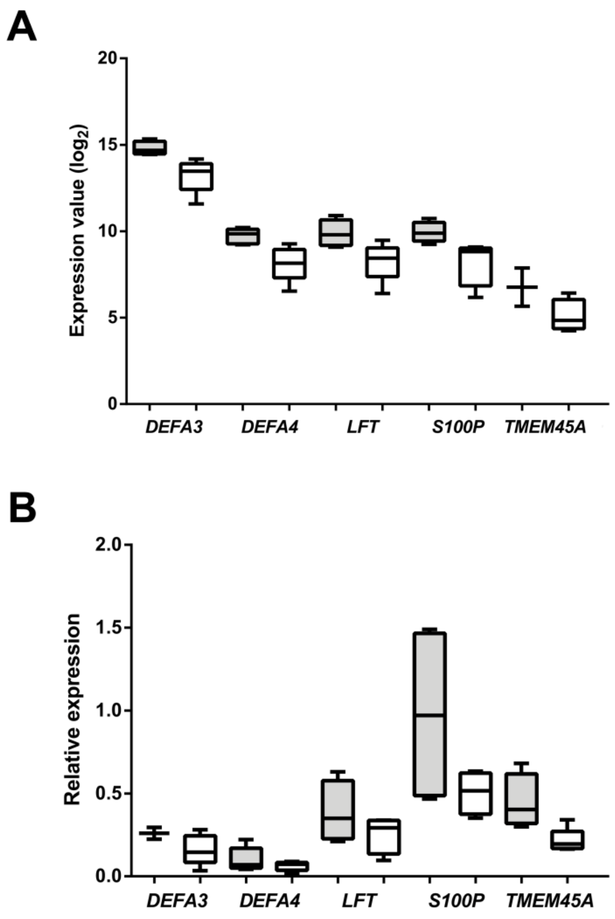

Figure S4. qPCR validation of DNA microarray data. In **A** the boxplots representing DNA microarray expression values of five selected genes in DS (gray) and CT (white) groups. In **B** qPCR expression fold changes boxplots of DS and CT groups for the same genes. Values for DS group indicate upregulation.

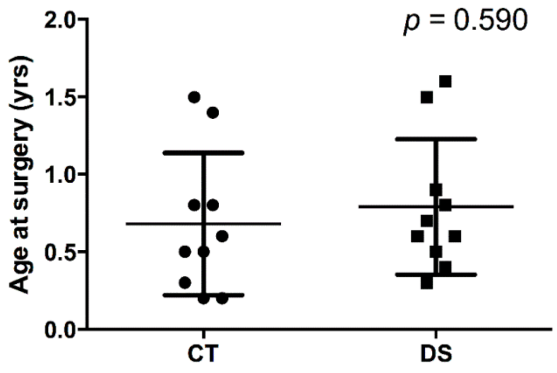

Figure S5. Patients' age at surgery. Scatter-plot of age at surgery (in years) of subjects with Down Syndrome and karyotypically normal control subjects and t-test p-value.

Table S1: Number of genes per community in DE networks

Communities	Number of genes	
	CT-DE Network	DS-DE Network
A	41	55
B	40	52
C	39	50
D	30	38
E	26	32
F	25	30
G	18	18
H	14	14
I	3	11

Table S2: Number of genes per community in CO networks

	Number of genes	
**Communities**	**CT- CO Network**	**DS-CO Network**
A	404[a]	587[a]
B	400[a]	533[a]
C	397[a]	436[a]
D	293[a]	436[a]
E	290[a]	393[a]
F	248[a]	316[a]
G	235[a]	312[a]
H	226[a]	289[a]
I	213[a]	252
J	188	199
K	180[a]	189[a]
L	165[a]	172[a]
M	152	145
N	147	119
O	132	105
P	124	100
Q	117[a]	95
R	106[a]	82
S	97	70[a]
T	95	69
U	92[a]	56
V	83	54
W	75	53

X	35	51
Y	32	48[a]
Z	30	46
A'	21	14
B'	-	8
C'	-	8
D'	-	7
E'	-	7
F'	-	6

[a]Communities harboring at least one high hierarchy gene and included in the coarse-grained community structure (CGCS) diagrams showed in Figure 4.

Table S3: Differentially expressed micro RNAs

microRNA	Mean	Mean	*p* value	fold (DS/CT)
	DS group	CT group		
**Up-regulated in DS group**				
hsa-miR-548d-5p	5.771	3.265	0.007	1.768
hsa-miR-196a-5p	4.847	3.071	0.040	1.578
hsa-miR-449a	3.470	1.990	0.028	1.744
hsa-miR-563	5.820	4.401	0.050	1.323
hsa-miR-30b-3p	2.811	1.665	0.035	1.688
hsa-miR-609	3.499	2.358	0.029	1.484
**Down-regulated in DS group**				
hsa-miR-548am-5p	2.528	3.526	0.034	0.717
hsa-miR-301a-3p	1.199	2.316	0.049	0.518
hsa-miR-129-2-3p	1.909	3.464	0.037	0.551
hsa-miR-139-5p_v18.0	0.886	2.667	0.023	0.332
hsa-let-7f-1-3p	5.290	7.083	0.041	0.747
hsa-miR-602	7.426	9.674	0.044	0.768
hsa-miR-484	4.532	6.849	0.015	0.662
hsa-miR-200b-5p	2.395	5.195	0.018	0.461
hsa-let-7b-3p	3.866	6.775	0.016	0.571
hsa-miR-1267	2.588	5.593	0.006	0.463
hsa-miR-191-3p	10.223	13.346	0.032	0.766
hsa-miR-204-5p	4.519	7.983	0.035	0.566
hsa-miR-486-5p	2.616	6.393	0.005	0.409
hsa-miR-127-3p	3.282	7.107	0.025	0.462
hsa-miR-550a-5p	6.955	10.836	0.043	0.642

hsa-miR-296-5p	4.230	8.499	0.010	0.498
hsa-miR-361-3p	3.714	8.529	0.009	0.436
hsa-miR-766-3p	13.347	18.531	0.046	0.720
hsa-miR-625-5p	9.784	15.070	0.043	0.649
hsa-miR-149-5p	8.622	14.136	0.018	0.610
hsa-miR-497-5p	5.445	11.516	0.019	0.473
hsa-miR-1249	6.347	14.024	0.007	0.453
hsa-miR-197-3p	23.434	32.666	0.035	0.717
hsa-miR-99b-5p	7.604	16.944	0.024	0.449
hsa-miR-30d-5p	15.219	26.102	0.035	0.583
hsa-miR-140-3p	16.540	27.855	0.032	0.594
hsa-miR-193b-3p	10.971	22.687	0.028	0.484
hsa-miR-424-3p	8.870	20.677	0.033	0.429
hsa-miR-125a-5p	13.624	26.490	0.016	0.514
hsa-miR-145-5p	13.777	27.115	0.016	0.508
hsa-miR-1280_v18.0	20.575	34.153	0.024	0.602
hsa-miR-331-3p	16.576	30.223	0.019	0.548
hsa-miR-151a-5p	16.946	35.105	0.018	0.483
hsa-miR-23b-3p	32.651	54.707	0.045	0.597
hsa-miR-455-3p	12.723	35.789	0.003	0.356
hsa-miR-324-3p	39.682	69.553	0.046	0.571
hsa-miR-200c-3p	52.551	88.058	0.034	0.597
hsa-miR-30c-5p	61.840	100.202	0.044	0.617
hsa-miR-1274b_v16.0	30.730	76.706	0.026	0.401
hsa-miR-29a-3p	52.271	120.050	0.030	0.435
hsa-miR-100-5p	75.757	153.017	0.031	0.495
hsa-miR-205-5p[a]	230.549	469.303	0.011	0.491
hsa-miR-720_v18.0[a]	309.105	575.821	0.034	0.537

hsa-miR-15b-5p[a]	456.515	737.685	0.040	0.619
hsa-miR-181a-5p[a]	478.509	827.574	0.037	0.578
hsa-miR-125b-5p[a]	663.695	1123.982	0.017	0.590
hsa-miR-150-5p[a]	531.064	1256.764	0.025	0.423

[a]microRNAs abundantly expressed in DS and CT groups.
