# Supplementary material for: Modular transcriptional repertoire and MicroRNA target analyses characterize genomic dysregulation in the thymus of Down syndrome infants
Source: Oncotarget. 2016 Feb 1;7(7):7497–533. doi: 10.18632/oncotarget.7120 (PMC4884935; doi:10.18632/oncotarget.7120)
Supplement: Supplementary file 2 [file oncotarget-07-7497-s002.pdf]

Table S4: Functional description of interactome nodes linked with HH genes produtcs in CT-DE network

CT-DE CGN Comm	HH gene product	Protein-related function	Interacting protein in 1st level	Protein-realted function	Biological function	Node shape (border color)
A	CASP4	apoptosis	GCN1L1	Translation factor activity, nucleic acid binding; regulation of translation	cell process	circle
			PSMD6	ATP-dependent proteolysis	cell process	circle
			CDK2	ATP binding; histone kinase activity; cyclin-dependent protein kinase activity	cell process/ATP binding	circle
			MDN1	ATPase binding; protein folding	cell process/ATP binding	circle
			MTHFD1L	ATP binding; protein homodimerization activity	cell process/ATP binding	circle
			SMC1A	ATP binding; chromatin binding; microtubule motor activity	cell process/ATP binding	circle
			SNRNP200	ATP binding; helicase activity	cell process/ATP binding	circle
			EIF2S3	GTP binding; GTPase activity; translation initiation factor activity	cell process/binding	circle
			HEATR2	Binding	cell process/binding	circle
			MSH6	ADP binding; chromatin binding	cell process/binding	circle
			SRPRB	GTP binding	cell process/binding	circle
			SSR4	Calcium ion binding; gene expression	cell process/binding/calcium ion binding	circle (light blue border)
			SMC2	DNA recombination; DNA repair; cell cycle	cell process/cell cycle	circle
			SMC4	Mitotic chromosome condensation; mitotic sister chromatid segregation; cell cycle	cell process/cell cycle	circle
			DCTN1	Motor activity; signaling protein activity involved in unfolded protein response; cell death	cell process/cell death	circle
			SMC3	ATP binding; microtubule motor activity	cell process/cytoskeleton	circle
			MYCBPAP	Protein binding; cell differentiation	cell process/differentiation	circle
			TMEM147	Endoplasmic reticulum	cell process/endoplasmic reticulum	circle
			COPE	COPI coating of Golgi vesicle; intra-Golgi vesicle-mediated transport	cell process/Golgi apparatus	circle
			VDAC1	Porin activity	cell process/ion transport	circle
			VDAC3	Porin activity	cell process/ion transport	circle
			UMPS	Lyase activity	cell process/lyase activity	circle
			CCDC59	Protein binding; regulation of transcription, DNA-dependent	cell process/protein binding	circle
			CTNNBIP1	Beta-catenin binding; negative regulation of Wnt receptor signaling pathway	cell process/signaling	circle
			PTPLAD1	Rac protein signal transduction; I-kappaB kinase/NF-kappaB cascade	cell process/signaling	circle
			HIGD1A	Response to stress	cell process/stress response	circle
			LSM10	Histone pre-mRNA DCP binding; RNA splicing	cell process/transcription	circle
			SNRPA1	RNA binding; RNA splicing	cell process/transcription	circle
			NUP93	Glucose transport; cytokine-mediated signaling pathway	cell process/transport	circle
			SLC25A1	Citrate transmembrane transporter activity; cellular lipid metabolic process	cell process/transport	circle
			SLC25A22	L-glutamate transmembrane transporter activity	cell process/transport	circle
			UBXN1	ATPase binding; polyubiquitin binding	ubiquitination	triangle
A	COG1	Golgi apparatus	COG4	Protein binding; Golgi vesicle prefusion complex stabilization; protein transport	cell process/Golgi apparatus	circle
A	MRPS6	mitochondrial protein	ESR1	Chromatin binding; estrogen receptor activity; estrogen response element binding	cell process/binding	circle
			C1ORF103	Receptor activity	cell process/signaling	circle
			KIAA1377	Protein binding	cell process/binding	circle
			RIF1	Protein binding; cytoskeleton	cell process/cytoskeleton	circle
A	RILPL2	MHC-II presentation	TUBB3	GTP binding	cell process/binding	circle
			TUBB4	MHC class I protein binding; cytoskeleton; natural killer cell mediated cytotoxicity	cell process/antigen presentation	circle (orange border)
			HEXDC	Carbohydrate metabolic process; hydrolase activity, hydrolyzing O-glycosyl compounds	cell process/hydrolase activity	circle
A	BTG3	T-cell development	CNOT3	Protein binding; nuclear-transcribed mRNA catabolic process	cell process/protein binding	circle
			E2F1	DNA binding; sequence-specific DNA binding transcription factor activity	cell process/transcription	circle
			ATXN1	DNA binding; RNA processing	cell process/transcription	circle
A	ARHGAP1	T-cell development	RHOC	GTPase activity; negative regulation of I-kappaB kinase/NF-kappaB cascade	GTPase activity/signaling	circle (yellow border)
			CDC42	GTP binding; GTPase activity; Thymopoise	cell process/T-cell differentiation	circle (light blue border)
			RHOA	GTP binding; GTPase activity; actin cytoskeleton organization	cytoskeleton	diamond
A	RNF11	TGF-beta signaling	UBQLN4	Polyubiquitin binding; regulation of proteasomal ubiquitin-dependent protein catabolic process	ubiquitination	triangle
			IKBKG	I-kappaB kinase/NF-kappaB cascade; T-cell receptor signaling pathway	cell process/signaling	circle
B	CEACAM1	T-cell development	CLEC4M	ICAM-3 receptor activity; peptide antigen binding; antigen processing and presentation; cell-cell recognition	cell process/antigen presentation	circle (orange border)
			SHC1	Ephrin receptor binding; Ras protein signal transduction; MAPK cascade	cell process/signaling	circle
C	HSPA13	apoptosis	UBQLN1	Kinase binding; apoptotic process	apoptosis	vee
			UBQLN4	Polyubiquitin binding; regulation of proteasomal ubiquitin-dependent protein catabolic process	ubiquitination	triangle
C	CD59	Hassal´s corpuscles	CFTR	PDZ domain binding; ATP-binding; phosphorylation-dependent chloride channel activity	cell process/ion transport	circle
			GRB2	SH3/SH2 adaptor activity; T-cell costimulation	cell process/T-cell development	circle (light blue border)
			EGFR	ATP binding; actin filament binding; nitric-oxide synthase regulator activity	apoptosis	vee
			SMAD4	DNA binding; chromatin DNA binding	cell process/transcription	circle
C	CGN	Hassal´s corpuscles	YWHAH	Actin binding	actin/binding	diamond
			SFN	Negative regulation of cell proliferation; apoptotic process	apoptosis	vee

			TJP1	Calmodulin binding; apoptotic process	apoptosis	vee
			ACTB	ATP binding; nitric-oxide synthase binding	cell process/ATP binding	circle
			YWHAB	Enzyme binding	cell process/binding	circle
			YWHAG	Insulin-like growth factor receptor binding	cell process/binding	circle
			YWHAZ	Protein binding	cell process/binding	circle
			ESR2	DNA binding; sequence-specific DNA binding transcription factor activity	cell process/transcription	circle
D	MORC3	Epigenetic regulator	HFLX	Official symbol: GTPBP6. GTP binding protein 6 (putative)	cell process/protein binding	circle
			DYNLL1	Actin cytoskeleton organization; activation of pro-apoptotic gene products	actin/cytoskeleton	diamond
D	DLG3	T-cell development	ERBB4	ATP binding; apoptotic process; epidermal growth factor receptor binding	apoptosis	vee
			GRIN2B	Beta-catenin binding; cell adhesion molecule binding	cell process/adhesion	circle
			LRFN2	Cell junction	cell process/adhesion	circle
			NLGN1	Neurexin family protein binding; cell adhesion molecule binding	cell process/adhesion	circle
			DGKI	ATP binding; diacylglycerol kinase activity	cell process/ATP binding	circle
			DGKZ	ATP binding; diacylglycerol kinase activity; cell migration	cell process/ATP binding	circle
			KLHDC3	Chromatin binding	cell process/binding	circle
			ANXA1	Calcium ion binding	cell process/binding/calcium ion binding	circle (light blue border)
			S100A3	Calcium ion binding	cell process/binding/calcium ion binding	circle (light blue border)
			ATP2B4	Calmodulin binding	cell process/calmodulin binding	circle (light blue border)
			CAMK2A	Calmodulin binding; positive regulation of NF-kappaB transcription factor activity	cell process/calmodulin binding	circle (light blue border)
			GRIN1	Calmodulin binding;	cell process/calmodulin binding	circle (light blue border)
			CRIPT	PDZ domain binding; cytoplasmic microtubule organization;	cell process/cytoskeleton	circle
			KRT31	Structural constituent of cytoskeleton	cell process/cytoskeleton	circle
			KRT34	Structural molecule activity; intermediate filament	cell process/cytoskeleton	circle
			KRT35	Structural molecule activity; intermediate filament	cell process/cytoskeleton	circle
			KRT82	Structural constituent of epidermis; keratin filament	cell process/cytoskeleton	circle
			KRT85	Structural molecule activity; keratin filament	cell process/cytoskeleton	circle
			GDA	Guanine deaminase activity	cell process/deaminase activity	circle
			GRIN2A	Calcium channel activity; cell adhesion molecule binding	cell process/ion transport	circle
			GRIN2C	Extracellular-glutamate-gated ion channel activity	cell process/ion transport	circle
			GRIN2D	Extracellular-glutamate-gated ion channel activity	cell process/ion transport	circle
			KCNA1	Potassium channel activity	cell process/ion transport	circle
			SSTR2	G-protein coupled receptor activity	cell process/signaling	circle
			HIST1H2BC	DNA binding; defense response to bacterium	cell process/transcription	circle
			ABCA1	ATP binding; cholesterol transporter activity	cell process/transport	circle
			CUL2	Ubiquitin protein ligase binding; induction of apoptosis by intracellular signals	ubiquitination	triangle
D	ARHGEF4	T-cell development	HLA-B	MHC class I receptor activity; antigen processing and presentation of peptide antigen via MHC class I	cell process/antigen presentation	circle (orange border)
			RAC1	T-cell costimulation; actin filament polymerization	cell process/T-cell costimulation	circle (light blue border)
E	GSTO1	stress response	UBA5	ATP binding	cell process/ATP binding	circle
			KIAA1377	Protein binding	cell process/binding	circle
			EEF1G	Protein binding; translation elongation factor activity	cell process/protein binding	circle
			C7ORF64	Official symbol: RBM48. RNA binding	cell process/transcription	circle
			SETDB1	DNA binding; regulation of transcription, DNA-dependent	cell process/transcription	circle
			KAT5	Histone acetyltransferase activity; chromatin modification	cell process/transferase activity	circle
			TRAF6	Histone deacetylase binding; ubiquitin-protein ligase activity	ubiquitination	triangle
E	APP	cell process	ACTB	ATP binding; nitric-oxide synthase binding	cell process/ATP binding	circle
			YWHAZ	Protein binding	cell process/binding	circle
			SHC1	Ephrin receptor binding; Ras protein signal transduction; MAPK cascade	cell process/signaling	circle
			GRB2	SH3/SH2 adaptor activity; T-cell costimulation	cell process/T-cell development	circle (light blue border)
F	FBXL5	Thymic microenviroment	RPOB	Official symbol: POLR1B. Transcription of ribosomal RNA (rRNA) genes and production of rRNA	cell process/transcription	circle
			MYB	DNA binding; chromatin remodeling; regulation of histone H3-K9 methylation	cell process/transcription	circle
F	MCM3AP	cell proliferation	APC	T-cell differentiation in thymus; microtubule binding	cell process/T-cell differentiation	circle (light blue border)
			SMAD9	Transforming growth factor beta receptor; pathway-specific cytoplasmic mediator activity	cell process	circle
			CELSR3	G-protein coupled receptor activity; cell adhesion; calcium ion binding	cell process/binding/calcium ion binding	circle (light blue border)
			CLSTN1	Calcium ion binding; cell adhesion	cell process/binding/calcium ion binding	circle (light blue border)
			CRMP1	Dihydropyrimidinase activity; cytoskeleton	cell process/cytoskeleton	circle
			TMEM216	Cilium morphogenesis; cytoskeleton	cell process/cytoskeleton	circle
			SDHA	Electron carrier activity; oxidation-reduction process	cell process/electon transport	circle
			RAC1	T-cell costimulation; actin filament polymerization	cell process/T-cell costimulation	circle (light blue border)
			CDC42	GTP binding; GTPase activity; Thymopoise	cell process/T-cell differentiation	circle (light blue border)
			SMAD3	Beta-catenin binding; chromatin DNA binding	cell process/transcription	circle
			NUDT5	ADP-ribose diphosphatase activity	cell process/transferase activity	circle
			ENY2	Transcription coactivator activity; chromatin modification; histone deubiquitination	ubiquitination	triangle
F	USO1	ER-golgi	ARF6	GTPase activity; apoptotic process	apoptosis	vee
			GAK	ATP binding; cell junction; heat shock protein binding	cell process/ATP binding	circle

			C1ORF94	Protein binding	cell process/binding	circle
			ASD	Official symbol: ARSD. Member of the sulfatase family; correct composition of bone and cartilage matrix	cell process/cytoskeleton	circle
			PTP4A3	Hydrolase activity; early endosome	cell process/hydrolase activity	circle
			GABPB1	GA-binding protein transcription factor, beta subunit	cell process/transcription	circle
			RSGA	Official symbol: ZNF24. Regulation of transcription, DNA-dependent	cell process/transcription	circle
			GSTK1	Glutathione transferase activity	cell process/transferase activity	circle
F	TBC1D9B	autophagy	MAX	Sequence-specific DNA binding transcription factor activity	cell process/transcription	circle
G	HSPA1A	antigen presentation	EGFR	ATP binding; actin filament binding; nitric-oxide synthase regulator activity	apoptosis	vee
			ESR1	Chromatin binding; estrogen receptor activity; estrogen response element binding	cell process/binding	circle
			YWHAB	Enzyme binding	cell process/binding	circle
			YWHAG	Insulin-like growth factor receptor binding	cell process/binding	circle
			YWHAZ	Protein binding	cell process/binding	circle
			CFTR	PDZ domain binding; ATP-binding; phosphorylation-dependent chloride channel activity	cell process/ion transport	circle
			ATXN1	DNA binding; RNA processing	cell process/transcription	circle
			ESR2	DNA binding; sequence-specific DNA binding transcription factor activity	cell process/transcription	circle
			SMAD3	Beta-catenin binding; chromatin DNA binding	cell process/transcription	circle
G	ZNHIT3	transcription	ESR1	Chromatin binding; estrogen receptor activity; estrogen response element binding	cell process/binding	circle
			NHP2L1	RNA binding; RNA splicing	cell process/transcription	circle
G	YIPF2	ER-golgi	SMC	Official symbol: DYM. Normal skeletal development and brain function	cell process/development	circle
			YIPF6	Endoplasmic reticulum	cell process/endoplasmic reticulum	circle
