# Supplementary material for: Modular transcriptional repertoire and MicroRNA target analyses characterize genomic dysregulation in the thymus of Down syndrome infants
Source: Oncotarget. 2016 Feb 1;7(7):7497–533. doi: 10.18632/oncotarget.7120 (PMC4884935; doi:10.18632/oncotarget.7120)
Supplement: Supplementary file 3 [file oncotarget-07-7497-s003.pdf]

Table S5: Functional description of interactome nodes linked with HH genes produtcs in DS-DE network

DS-DE CGN						
**Comm**	**HH gene product**	**Protein-related function**	**Interacting protein in 1st level**	**Protein-related function**	**Biological function**	**Node shape (border color)**
A	CYB5R1	Oxidative stress	UBQLN4	Polyubiquitin binding; regulation of proteasomal ubiquitin-dependent protein catabolic process	ubiquitination	triangle
A	ATP6AP1	macroautophagy	ANG	DNA binding; actin binding; actin filament polymerization	cell process/transcription	circle
			HBA1	Hemoglobin, alpha 1	cell process/hemoglobin	circle
A	PTRH1	hydrolase activity	PRNP	Chaperone binding; microtubule binding	cell process/protein binding	circle
A	HOMER2	T-cell development	APP	Amyloid precursor protein	cell process	circle
A	MPST	antioxidant protein	CHM	GTPase activator activity; Rab GTPase binding;	GTPase activity	circle (yellow border)
B	ANXA9	binding protein	ARHGEF10L	Rho GTPase activator activity	GTPase activity	circle (yellow border)
			NDUFA4L2	NADH dehydrogenase [ubiquinone] 1 alpha subcomplex subunit 4-like 2	cell process/electon transport	circle
B	PIP	signaling	DYNLL1	Actin cytoskeleton organization; activation of pro-apoptotic gene products	actin/cytoskeleton	diamond
			GABARAPL1	GABA receptor binding; beta-tubulin binding; autophagic vacuole	autophagy	square
			CEP290	Involved in ciliary vesicle formation (Kobayashi et al, 2014)	cell process	circle
			WBP5	WW domain binding	cell process/binding	circle
			SGK1	ATP binding; protein serine/threonine kinase activity	cell process/signaling	circle
			BUD13	Nuclear mRNA splicing, via spliceosome; RES complex	cell process/transcription	circle
			ESR2	DNA binding; sequence-specific DNA binding transcription factor activity	cell process/transcription	circle
			RAB5C	GTP binding; GTPase activity; endosome organization;protein transport	GTPase activity	circle (yellow border)
			UBA1	ATP binding; nucleotide binding; cell death; ubiquitin- proteasome system	ubiquitination	triangle
			USP25	SUMO binding; cysteine-type peptidase activity; ubiquitin thiolesterase activity	ubiquitination	triangle
			USPL1	Ubiquitin thiolesterase activity	ubiquitination	triangle
B	NLRP12	T-cell development	DLG4	Protein C-terminus binding; scaffold protein binding	cell process/protein binding	circle
C	CASP4	Apoptosis; thymopoiesis	HEATR2	Binding	cell process/binding	circle
			CCDC59	Protein binding; regulation of transcription, DNA-dependent	cell process/protein binding	circle
			CDK2	ATP binding; histone kinase activity; cyclin-dependent protein kinase activity	cell process/ATP binding	circle
			COPE	COPI coating of Golgi vesicle; intra-Golgi vesicle-mediated transport	cell process/Golgi apparatus	circle
			CTNNBIP1	Beta-catenin binding; negative regulation of Wnt receptor signaling pathway	cell process/signaling	circle
			DCTN1	Motor activity; signaling protein activity involved in unfolded protein response; cell death	cell process/cell death	circle
			EIF2S3	GTP binding; GTPase activity; translation initiation factor activity	cell process/binding	circle
			GCN1L1	Translation factor activity, nucleic acid binding; regulation of translation	cell process	circle
			HIGD1A	Response to stress	cell process/stress response	circle
			LSM10	Histone pre-mRNA DCP binding; RNA splicing	cell process/transcription	circle
			MDN1	ATPase binding; protein folding	cell process/ATP binding	circle
			MSH6	ADP binding; chromatin binding	cell process/binding	circle
			MTHFD1L	ATP binding; protein homodimerization activity	cell process/ATP binding	circle
			MYCBPAP	Protein binding; cell differentiation	cell process/differentiation	circle
			NUP93	Glucose transport; cytokine-mediated signaling pathway	cell process/transport	circle
			PSMD6	ATP-dependent proteolysis	cell process	circle
			PTPLAD1	Rac protein signal transduction; I-kappaB kinase/NF-kappaB cascade	cell process/signaling	circle
			SLC25A1	Citrate transmembrane transporter activity; cellular lipid metabolic process	cell process/transport	circle
			SLC25A22	L-glutamate transmembrane transporter activity	cell process/transport	circle
			SMC1A	ATP binding; chromatin binding; microtubule motor activity	cell process/ATP binding	circle
			SMC2	DNA recombination; DNA repair; cell cycle	cell process/cell cycle	circle
			SMC3	ATP binding; microtubule motor activity	cell process/cytoskeleton	circle
			SMC4	Mitotic chromosome condensation; mitotic sister chromatid segregation; cell cycle	cell process/cell cycle	circle
			SNRNP200	ATP binding; helicase activity	cell process/ATP binding	circle
			SNRPA1	RNA binding; RNA splicing	cell process/transcription	circle
			SRPRB	GTP binding	cell process/binding	circle
			SSR4	Calcium ion binding; gene expression	cell process/binding/calcium ion binding	circle (light blue border)
			TMEM147	Endoplasmic reticulum	cell process/endoplasmic reticulum	circle
			UBXN1	ATPase binding; polyubiquitin binding	ubiquitination	triangle
			UMPS	Lyase activity	cell process/lyase activity	circle
			VDAC1	Porin activity	cell process/ion transport	circle
			VDAC3	Porin activity	cell process/ion transport	circle
C	TRIM39	antiapoptosis	PDLIM5	Actin binding	actin/binding	diamond
			PLCD4	Member of the delta class of phospholipase C enzymes	cell process	circle
			ABL1	ATP binding; DNA binding; SH3 domain binding; actin monomer binding	cell process/ATP binding	circle
			FYN	ATP binding; ephrin receptor binding; protein tyrosine kinase activity	cell process/ATP binding	circle
			NCK1	Actin filament organization	cell process/cytoskeleton	circle
			UBE2V1	MyD88-dependent toll-like receptor signaling pathway; T-cell receptor signaling pathway; cell differentiation	cell process/inflammation	circle
			CRK	SH2 domain binding; SH3/SH2 adaptor activity; activation of MAPKK activity; involved in phagocytosis of apoptotic cells and cell motility	cell process/phagocytosis	circle

			GRB2	SH3/SH2 adaptor activity; T-cell costimulation	cell process/T-cell development	circle (light blue border)
			DCUN1D1	Protein binding; ubiquitin ligase complex	ubiquitination	triangle
			HIP2	UBE2K; belongs to the ubiquitin-conjugating enzyme family	ubiquitination	triangle
			TRIM17	Metal ion binding; ubiquitin-protein ligase activity; protein autoubiquitination	ubiquitination	triangle
			UBE2D1	ATP binding; ubiquitin-protein ligase activity; BMP signaling pathway	ubiquitination	triangle
			UBE2D2	ATP binding; acid-amino acid ligase activity; ubiquitin protein ligase binding	ubiquitination	triangle
			UBE2D3	ATP binding; ubiquitin-protein ligase activity; BMP signaling pathway	ubiquitination	triangle
			UBE2D4	ATP binding; acid-amino acid ligase activity; ubiquitin protein ligase binding	ubiquitination	triangle
			UBE2E1	ATP binding; ubiquitin-protein ligase activity; ISG15-protein conjugation	ubiquitination	triangle
			UBE2E2	ATP binding; ubiquitin-protein ligase activity; ISG15-protein conjugation	ubiquitination	triangle
			UBE2E3	ATP binding; acid-amino acid ligase activity; ubiquitin protein ligase binding	ubiquitination	triangle
			UBE2L3	ATP binding; acid-amino acid ligase activity; ubiquitin protein ligase binding	ubiquitination	triangle
			UBE2W	ATP binding; ubiquitin-protein ligase activity	ubiquitination	triangle
			UBTD1	Ubiquitin family protein	ubiquitination	triangle
C	FOXJ3	cell cycle	MED23	Protein binding; transcription coactivator activity;	cell process/protein binding	circle
C	FBXL5	iron homeostasis	RPOB	Official symbol: POLR1B. Transcription of ribosomal RNA (rRNA) genes and production of rRNA	cell process/transcription	circle
			MYB	DNA binding; chromatin remodeling; positive regulation of histone H3-K9 methylation	cell process/transcription	circle
C	PGRMC1	adaptor protein for steroid receptors	IKBKE	ATP binding; IkappaB kinase activity; NF-kappaB-inducing kinase activity	apoptosis	vee
			VHL	Transcription factor binding; anti-apoptosis	apoptosis/anti-apoptosis	vee
			ATG5	protein binding; autophagic vacuole assembly; autophagy	autophagy	square
			PGRMC1	Adaptor protein for steroid receptors	cell process	circle
			HLA-B	MHC class I receptor activity; antigen processing and presentation of peptide antigen via MHC class I	cell process/antigen presentation	circle (orange border)
			ABCD3	ATP binding; ATPase activity, coupled to transmembrane movement of substances; protein self-association	cell process/ATP binding	circle
			HEATR2	Binding	cell process/binding	circle
			EFHD1	Calcium ion binding	cell process/binding/calcium ion binding	circle (light blue border)
			MTNR1A	Melatonin receptor activity; G-protein signaling, coupled to cyclic nucleotide second messenger; circadian rhythm	cell process/circadian rhythm	circle
			SFXN1	Iron ion transport; erythrocyte differentiation	cell process/ion transport	circle
			TIMP2	Enzyme activator activity; negative regulation of cell proliferation; regulation of MAPK cascade	cell process/proliferation	circle
			MCC	Protein binding; Wnt receptor signaling pathway	cell process/signaling	circle
			NME2	ATP binding; protein histidine kinase activity	cell process/signaling	circle
			PAK2	ATP binding; nucleotide binding; protein serine/threonine kinase activity	cell process/signaling	circle
			MPG	DNA binding; alkylbase DNA N-glycosylase activity; base-excision repair	cell process/transcription	circle
			DDOST	Dolichyl-diphosphooligosaccharide-protein glycotransferase activity	cell process/transferase activity	circle
			EIF1B	Regulation of translational initiation	cell process/translation	circle
			TRAF6	Histone deacetylase binding; ubiquitin-protein ligase activity	ubiquitination	triangle
C	PTPN12	T-cell development	GRB2	SH3/SH2 adaptor activity; T-cell costimulation	cell process/T-cell development	circle (light blue border)
C	NDUFS4	mitochondrial respiration chain	NDUFA2	NADH dehydrogenase (ubiquinone) activity; mitochondrial electron transport	mitochondrial respiratory chain	circle (light green border)
			SNCA	Hsp70 protein binding; alpha-tubulin binding; cysteine-type endopeptidase inhibitor activity involved in apoptotic process	apoptosis	vee
C	NDUFV3	mitochondrial respiration chain	NDUFA2	NADH dehydrogenase (ubiquinone) activity; mitochondrial electron transport	mitochondrial respiratory chain	circle (light green border)
			PHYHIP	Phytanoyl-CoA hydroxylase-interacting protein	cell process/binding	circle
C	NFU1	mitochondrial respiration chain	C4ORF14	NOA1, nitric oxide-associated protein 1; mitochondrial activity regulator	cell process/mitochondrial activity	circle
			SH3BP4	Protein binding; endocytosis	cell process/protein binding	circle
D	P2RY2	phagocytic clearance	SLC9A3R1	PDZ domain binding; beta-catenin binding; Wnt receptor signaling pathway	cell process/signaling	circle
D	DOK7	thymic development	GABARAPL2	Autophagy	autophagy	square
D	NFE2	transcription	DLG4	ATP binding; ephrin receptor binding; protein tyrosine kinase activity	cell process/ATP binding	circle
			PHYHIP	Phytanoyl-CoA hydroxylase-interacting protein	cell process/binding	circle
F	LAPTM4B	autophagy	PIK3R1	ErbB-3 class receptor binding; insulin receptor substrate binding	cell process/insulin receptor	circle
			ABCB1	ATP binding; ATPase activity	cell process/ATP binding	circle
F	C11ORF24	Golgi apparatus	CORE	Official symbol: RYR1. Encodes a ryanodine receptor; calcium release channel in the sarcoplasmic reticulum	cell process/ion transport	circle
F	ARHGAP1	T-cell development	RHOA	GTP binding; GTPase activity; actin cytoskeleton organization	GTPase activity	circle (yellow border)
			RHOC	GTPase activity; negative regulation of I-kappaB kinase/NF-kappaB cascade	GTPase activity/signaling	Circle (yellow border)
			CDC42	GTP binding; GTPase activity; Thymopoise	cell process/T-cell differentiation	circle (light blue border)
