# Supplementary material for: Modular transcriptional repertoire and MicroRNA target analyses characterize genomic dysregulation in the thymus of Down syndrome infants
Source: Oncotarget. 2016 Feb 1;7(7):7497–533. doi: 10.18632/oncotarget.7120 (PMC4884935; doi:10.18632/oncotarget.7120)
Supplement: Supplementary file 4 [file oncotarget-07-7497-s004.pdf]

Table S6: Functional description of interactome nodes linked with HH genes produtcs in CT-CO network

CT-CO CGN<br>Comm	HH gene product	Protein-related function	Interacting protein in 1st level	Protein-related function	Biological function	Node shape (border color)
A	SPTBN2	T-cell development	CDK1	ATP binding; protein kinase activity; histone kinase activity; cyclin-dependent protein kinase activity	cell process/ATP binding	circle
			CELSR3	G-protein coupled receptor activity; cell adhesion; calcium ion binding	cell process/binding/calcium ion binding	circle (light blue border)
			APC	T-cell differentiation in thymus; microtubule binding	cell process/T-cell differentiation	circle (light blue border)
			ESR2	DNA binding; sequence-specific DNA binding transcription factor activity	cell process/transcription	circle
B	ADNP2	cell survival	NFYC	Regulation of transcription; antigen processing and presentation; cellular lipid metabolic process	cell process/antigen presentation	circle (light blue border)
B	C18ORF25	T-cell development	UBE2I	SUMO ligase activity	ubiquitination	triangle
B	ERCC3	transcription	MYC	Cell cycle progression; apoptosis; cellular transformation; canonical Wnt signaling pathway	apoptosis	vee
			PSMC5	ATP binding; thyrotropin-releasing hormone receptor binding	cell process/ATP binding	circle
			AR	Androgen receptor activity; androgen binding	cell process/binding	circle
			GTF2H1	DNA repair; nucleotide-excision repair	cell process/transcription	circle
			SMAD2	Chromatin binding; SMAD binding	cell process/transcription	circle
B	HNRNPA0	antigen presentation	GABARAP	Apoptotic process; microtubule cytoskeleton organization	apoptosis	vee
			MYC	Cell cycle progression; apoptosis; cellular transformation; canonical Wnt signaling pathway	apoptosis	vee
			MAP1LC3A	Autophagic vacuole assembly; microtubule-associated protein	autophagy	square
B	SNAP23	MHC-I transport	FXR2	RNA binding	cell process/transcription	circle
			NAPA	SNARE complex disassembly; intracellular protein transport	cell process/protein transport	circle
B	TAPBP	antigen presentation	CAPR	Official symbol: CTNNA2. Hippo signaling pathway; single organismal cell-cell adhesion	cell process/signaling	circle
			PDIA3	Antigen processing and presentation of peptide antigen via MHC class I	cell process/antigen presentation	circle (light blue border)
			TAP1	Antigen processing and presentation of endogenous peptide antigen via MHC class I; positive regulation of T-cell mediated cytotoxicity	cell process/antigen presentation	circle (light blue border)
			TAP2	Antigen processing and presentation of endogenous peptide antigen via MHC class I; positive regulation of T-cell mediated cytotoxicity	cell process/antigen presentation	circle (light blue border)
			RPOB	Official symbol: POLR1B. Transcription of ribosomal RNA (rRNA) genes and production of rRNA	cell process/transcription	circle
B	TNK2	signaling	CDC42	GTP binding; GTPase activity; Thymopoise	cell process/T-cell differentiation	circle (light blue border)
			TNK2	Component of EGF receptor signaling complex; enhances thymic epithelial cell sustaining thymocyte differentiation	cell process/signaling	circle
C	EDEM3	T-cell related	ZWINT	Cell cycle; mitotic cell cycle checkpoint	cell process/cell cycle	circle
C	RAP1GDS1	T-cell related	CDC42	GTP binding; GTPase activity; Thymopoise	cell process/T-cell differentiation	circle (light blue border)
			RAC1	T-cell costimulation; actin filament polymerization	cell process/T-cell costimulation	circle (light blue border)
			MBIP	Histone H3 acetylation; protein kinase inhibitor activity	cell process/transcription	circle
			ZNF451	Regulation of transcription, DNA-dependent	cell process/transcription	circle
D	ARHGAP18	T-cell development	MPHOSPH6	RNA binding; nuclear exosome (RNase complex)	cell process/protein binding	circle
D	NDUFB4	mitochondrial respiration chain	MME	Endopeptidase activity; metalloendopeptidase activity	cell process/catalytic activity	circle
			SNCA	Hsp70 protein binding; cysteine-type endopeptidase inhibitor activity involved in apoptotic process	apoptosis	vee
E	BAG4	signaling	MAP1LC3A	Autophagic vacuole assembly; microtubule-associated protein	autophagy	square
			MAP1LC3B	Autophagy	autophagy	square
			GABARAP	Apoptotic process; microtubule cytoskeleton organization	apoptosis	vee
F	BCKDHB	metabolism	BCKDHA	Alpha-ketoacid dehydrogenase activity; metal ion binding	cell process/ion binding	circle
F	GLRX3	T-cell related	IKBKE	ATP binding; IkappaB kinase activity; NF-kappaB-inducing kinase activity	apoptosis	vee
			HEXDC	Carbohydrate metabolic process; hydrolase activity, hydrolyzing O-glycosyl compounds	cell process/hydrolase activity	circle
			FRA10AC1	Protein binding	cell process/protein binding	circle
			ZNF512B	Regulation of transcription, DNA-dependent	cell process/transcription	circle
F	PHF20	regulator of NF-κB	MYC	Cell cycle progression; apoptosis; cellular transformation; canonical Wnt signaling pathway	apoptosis	vee
			MYST1	Histone acetylase protein; myeloid cell differentiation	cell process/differentiation	circle
			WDR5	Histone acetyltransferase activity; chromatin modification	cell process/transcription	circle
			HIST1H3J	Member of the histone H3 family	cell process/transport	circle
			HIST1H4A	Member of the histone H4 family	cell process/transport	circle
			DLG4	Protein C-terminus binding; scaffold protein binding	cell process/protein binding	circle
G	INVS	cell migration	ZNF512B	Regulation of transcription, DNA-dependent	cell process/transcription	circle
			HIF1AN	NF-kappaB binding; oxidoreductase activity; iron ion binding	cell process/ion binding	circle
			PINX1	Negative regulation of cell proliferation; telomerase inhibitor activity	cell process/transcription	circle
H	DNAJB6	T-cell development	MYC	Cell cycle progression; apoptosis; cellular transformation; canonical Wnt signaling pathway	apoptosis	vee
H	LYPLA1	T-cell development	NS	Official symbol: KRAS. Fc-epsilon receptor signaling pathway; insulin receptor signaling pathway	cell process/signaling	circle
			IKBKE	ATP binding; IkappaB kinase activity; NF-kappaB-inducing kinase activity	apoptosis	vee
			VHL	transcription factor binding; anti-apoptosis	apoptosis/anti-apoptosis	vee
K	C7ORF25	mitochondrial activity	HEXDC	Carbohydrate metabolic process; hydrolase activity, hydrolyzing O-glycosyl compounds	cell process/hydrolase activity	circle
			FRA10AC1	Protein binding	cell process/protein binding	circle
			BRD4	Protein phosphorylation; histone acetylation; chromosome segregation	cell process/transcription	circle
			FZR1	Kinase activity; ubiquitin-dependent protein catabolic process	ubiquitination	triangle
L	HM13	antigen presentation	ABHD16A	Hydrolase activity; involved in some aspects of immunity	cell process/hydrolase activity	circle
			P4HB	Electron carrier activity; lipid metabolic process; cell redox homeostasis	cell process/electon transport	circle
Q	PIK3CD	T-cell related	TNPO2	Aliase: TNR2. intracellular protein transport; apoptosis	apoptosis	vee

	FAM195A	Family with sequence similarity 195, member A	cell process	circle
	PIK3R1	ErbB-3 class receptor binding; insulin receptor substrate binding	cell process/insulin receptor	circle
	ASCC2	Regulation of transcription, DNA-dependent	cell process/transcription	circle
	RALY	Nuclear mRNA splicing, via spliceosome	cell process/transcription	circle
	NCK1	Actin filament organization	cytoskeleton	diamond
