# Supplementary material for: Modular transcriptional repertoire and MicroRNA target analyses characterize genomic dysregulation in the thymus of Down syndrome infants
Source: Oncotarget. 2016 Feb 1;7(7):7497–533. doi: 10.18632/oncotarget.7120 (PMC4884935; doi:10.18632/oncotarget.7120)
Supplement: Supplementary file 5 [file oncotarget-07-7497-s005.pdf]

Table S7: Functional description of interactome nodes linked with HH genes produtcs in DS-CO network

DS-CO CGN<br>Comm	HH gene product	Protein-related function	Interacting protein in 1st level	Protein-related function	Biological function	Node shape (border color)
A	ZMYM1	ion binding	CTCF	Chromatin modification; maintenance of DNA methylation	cell process/transcription	circle
A	CHCHD3	mitochondrial activity	MYC	Cell cycle progression; apoptosis; cellular transformation; canonical Wnt signaling pathway	apoptosis	vee
			SNCA	Hsp70 protein binding; alpha-tubulin binding; cysteine-type endopeptidase inhibitor activity involved in apoptotic process	apoptosis	vee
			SERA	L-serine synthesis; cellular amino acid biosynthetic process	cell process/amino acid biosynthesis	circle
			ERH	Cell cycle	cell process/cell cycle	circle
			MAU2	Chromosome segregation; cell cycle	cell process/cell cycle	circle
			CCDC85B	Cell differentiation; cytoskeleton	cell process/cytoskeleton	circle
			RECQL4	ATP-dependent 3'-5' DNA helicase activity; zinc ion binding	cell process/ion binding	circle
			KRTAP4-12	Keratin filament	cell process/keratin	circle
A	CCNI	T-cell development	CEP63	Signal transduction in response to DNA damage; DNA damage checkpoint	cell process/signaling	circle
A	NCAPH2	T-cell development	NCAPD3	Mitotic chromosome condensation; cell cycle	cell process/cell cycle	circle
			NCAPG2	Inner cell mass cell proliferation; chromosome condensation	cell process/cell cycle	circle
			SMC2	DNA recombination; DNA repair; cell cycle	cell process/cell cycle	circle
			SMC4	Mitotic chromosome condensation; mitotic sister chromatid segregation; cell cycle	cell process/cell cycle	circle
			PA	Erythrocyte shape and mechanical property regulation; iron ion homeostasis; peptide cross-linking	cell process/cell shape	circle
A	MOBKL1A	T-cell development	MST1	Catalytic activity	cell process/catalytic activity	circle
			LATS1	Protein serine/threonine kinase activity; cell cycle	cell process/signaling	circle
			LATS2	Protein phosphorylation; hippo signaling cascade	cell process/signaling	circle
			STK38	Protein phosphorylation; protein modification process	cell process/transcription	circle
A	MIER3	transcription	HDAC2	Histone deacetylase activity (H3-K16 specific); chromatin binding	cell process/transcription	circle
C	TPM3	cytoskeleton	EIF1B	Regulation of translational initiation	cell process/transcription	circle
C	ATPIF1	mitophagy	CHD3	Zinc ion binding	cell process/ion binding	circle
			UBQLN4	Polyubiquitin binding; regulation of proteasomal ubiquitin-dependent protein catabolic process	ubiquitination	triangle
			PIK3CA	Protein serine/threonine kinase activity; insulin receptor substrate binding	cell process/signaling	circle
C	EXOSC9	T-cell development	GABARAP	Apoptotic process; microtubule cytoskeleton organization	apoptosis	vee
			GABARAPL1	GABA receptor binding; beta-tubulin binding; autophagic vacuole	autophagy	square
			DDX39B	ATP binding; helicase and hydrolase activity	cell process/ATP binding	circle
			ZFP36	C-C chemokine binding; RNA binding	cell process/binding	circle
			BAT1	ATP hydrolysis during pre-mRNA splicing	cell process/transcription	circle
			EXOSC10	3'-5' exonuclease activity; RNA processing	cell process/transcription	circle
			EXOSC2	3'-5'-exoribonuclease activity; positive regulation of cell growth	cell process/transcription	circle
			EXOSC3	3'-5'-exoribonuclease activity; RNA binding	cell process/transcription	circle
			EXOSC4	3'-5'-exoribonuclease activity; histone mRNA catabolic process	cell process/transcription	circle
			EXOSC7	3'-5'-exoribonuclease activity; RNA processing	cell process/transcription	circle
			UBE2I	SUMO ligase activity	ubiquitination	triangle
C	LRRFIP1	T-cell development	CHBCAT	Wnt Signaling Pathway; B Cell Receptor Signaling Pathway; adhesion	cell process/signaling	circle
			GRIP1	Androgen receptor binding; beta-catenin binding	cell process/binding	circle
			FLII	Actin cytoskeleton; actin binding protein	actin/cytosckeleton	diamond
C	RBM4	transcription	SMAD5	Ubiquitin protein ligase binding; signal transduction, TGFbeta recptor	ubiquitination	triangle
C	TAF1A	transcription	SET	Negative regulation of histone acetylation; apoptosis	apoptosis	vee
			TBP	Cell death; transcription factor binding	cell process/cell death	circle
			TAF1B	Transcription, DNA-dependent	cell process/transcription	circle
			TAF1D	Regulation of transcription, DNA-dependent	cell process/transcription	circle
D	PHF20L1	transcription	CALM1	Calcium-binding protein family	cell process/binding/calcium ion binding	circle (light blue border)
			MYST1	Histone acetylase protein; myeloid cell differentiation	cell process/differentiation	circle
			HIST1H3J	Member of the histone H3 family	cell process/transcription	circle
			HIST1H4A	Member of the histone H4 family	cell process/transcription	circle
E	DPP8	cell process	CXCL11	Immune response; chemotaxis; cell surface receptor signaling pathway	cell process/immune response	circle (light blue border)
			CXCL10	Chemokine activity; immune response; inflammatory response	cell process/immune response	circle (light blue border)
			CXCL12	Immune respose; chemokine activity; cell adhesion; cellular calcium ion homeostasis	cell process/immune response	circle (light blue border)
E	PHC1	T-cell development	HSPA8	ATP binding	cell process/ATP binding	circle
			PHC2	Metal ion binding; DNA binding	cell process/ion binding	circle
			CBX2	Component of the polycomb multiprotein complex; chromatin remodeling and modification of histones; cell differentiation	cell process/transcription	circle
			SFMBT1	Regulation of transcription, DNA-dependent	cell process/transcription	circle
			BMI1	Ubiquitin-protein ligase activity; embryonic skeletal system morphogenesis	ubiquitination	triangle
			CBX8	Histone ubiquitination	ubiquitination	triangle
			PCGF2	Histone acetylation; ubiquitin-protein ligase activity	ubiquitination	triangle
			UBE2I	SUMO ligase activity	ubiquitination	triangle
E	WHSC1	transcription	AR	Androgen receptor activity; androgen binding	cell process/binding	circle

			MDM2	Ubiquitin-protein ligase activity; DNA damage response; signal transduction by p53 class mediator resulting in cell cycle arrest	ubiquitination	triangle
F	HSPG2	thymus conduit	ALSD	Official symbol: PTGS2. Cyclooxygenase; prostaglandin biosynthesis	cell process/biosynthesis	circle
			ATN1	Cell death; cell migration	cell process/cell death	circle
			GRN	Cytokine activity; positive regulation of epithelial cell proliferation	cell process/cell proliferation	circle
			GRB2	SH3/SH2 adaptor activity; T-cell costimulation	cell process/T-cell development	circle (light blue border)
			GFI1B	Chromatin modification	cell process/transcription	circle
G	RPS2	transcription	GABARAP	Apoptotic process; microtubule cytoskeleton organization	apoptosis	vee
			MLH1	DNA damage response; signal transduction resulting in induction of apoptosis	apoptosis	vee
			MYC	Cell cycle progression; apoptosis; cellular transformation; canonical Wnt signaling pathway	apoptosis	vee
			GABARAPL1	GABA receptor binding; beta-tubulin binding; autophagic vacuole	autophagy	square
			GABARAPL2	Autophagy	autophagy	square
			MAP1LC3A	Autophagic vacuole assembly; microtubule-associated protein	autophagy	square
			ESR1	Chromatin binding; estrogen receptor activity; estrogen response element binding	cell process/binding	circle
			STAU1	Microtubule associated complex; cytoplasmic stress granule	cell process/cytoskeleton	circle
			MPP3	Signal transduction	cell process/signaling	circle
			ARRB1	Ubiquitin protein ligase binding; insulin-like growth factor receptor binding	ubiquitination	triangle
H	PRPF4B	T-cell development	PRPF4	Thymocyte survival	cell process/T-cell development	circle (light blue border)
K	MAP4	cell motility	SNCA	Hsp70 protein binding; alpha-tubulin binding; cysteine-type endopeptidase inhibitor activity involved in apoptotic process	apoptosis	vee
			MAP1LC3A	Autophagic vacuole assembly; microtubule-associated protein	autophagy	square
Y	ST13	cell migration	GABARAPL2	Autophagy	autophagy	square
			TNFRSF14	ubiquitin protein ligase binding	ubiquitination	triangle
			BAG1	chaperone binding; apoptotic process	apoptosis	vee
			TNF	cytokine activity; MAPK cascade	cell process/signaling	circle
			MAP3K3	ATP binding; MAPK cascade	cell process/signaling	circle
			IKBKE	ATP binding; IkappaB kinase activity	apoptosis	vee
			APOE	antioxidant activity; cholesterol transporter activity	cell process/cholesterol transport	circle
