# Supplementary material for: Modular transcriptional repertoire and MicroRNA target analyses characterize genomic dysregulation in the thymus of Down syndrome infants
Source: Oncotarget. 2016 Feb 1;7(7):7497–533. doi: 10.18632/oncotarget.7120 (PMC4884935; doi:10.18632/oncotarget.7120)
Supplement: Supplementary file 6 [file oncotarget-07-7497-s006.pdf]

Table S8: Primer sequences used for validation of gene expression by qPCR

Gene	Primer foward (5' - 3')	Primer reverse (5' - 3')	Product lenght (pb)
DEFA3	GACTGCTGTCTGCCCTCTCT	GCAGAATGCCCAGAGTCTTC	337
DEFA4	CCTTTGCATGGGATAAAAGC	CAACCATTTCCTGTAGCTCTCA	298
LFT	GATAAGGTGGAACGCCTGAA	GAATGGCTGAGGCTTTCTTG	312
S100P	TACCAGGCTTCCTGCAGAGT	GGCTCAGCCTAGGGGAATAA	256
TMEM45A	GCAAAAGCGAACCTGCTATC	AACGAGGCCTGTCAGAAAGA	400
GAPDH	ACCACAGTCCATGCCATCAC	TCCACCACCCTGTTGCTGTA	452
